# FOCUS: A Four‐In‐One Consolidated Unison Strain Sensor with Enhanced Sensitivity

**DOI:** 10.1002/advs.202519613

**Published:** 2026-01-27

**Authors:** Zimeng Wang, Ruiran Li, Bowen Yang, Zhuofei Peng, Muyang Jing, Yu Gu, Lixue Tang

**Affiliations:** ^1^ School of Biomedical Engineering Capital Medical University Beijing China; ^2^ Beijing Key Laboratory of Intelligent Diagnosis Technology and Equipment for Optic Nerve‐Related Eye Diseases Beijing China

**Keywords:** 3D Structure, flexible strain sensor, liquid metal, sensitivity amplification, wearable device

## Abstract

Liquid metal strain sensors possess outstanding stretchability, low modulus, and excellent repeatability, making them ideal for large‐deformation measurements. However, traditional liquid metal sensors inherently suffer from low sensitivity, which severely limits their capability in detecting subtle deformations. To achieve enhanced sensitivity, we have proposed a new paradigm: FOCUS, a Four‐in‐One Consolidated Unison Strain Sensor specifically created for microscale biomechanical deformation. In this work, four liquid metal strain sensors were printed on an elastic planar substrate and subsequently folded to overlap at the same location. Upon stretching, two of the sensors exhibited an increase in resistance, whereas the other two displayed a greater decrease. By leveraging this complementary response within a Wheatstone bridge configuration, FOCUS exhibits a fivefold enhancement in sensitivity compared to a single liquid metal sensor, while simultaneously maintaining signal stability against wide temperature range. FOCUS can accurately capture strains ranging from centimeters to micrometers, resolution reaches 25 micrometers. To demonstrate the powerful performance of FOCUS, we used it to perform periocular micro‐motion tracking and potential deformation monitoring of other human joints.

## Graphical abstract

Through a folding transformation that stacks four printed liquid metal sensors into a unified 3D orthogonal construct, FOCUS harnesses complementary bidirectional resistance responses within a Wheatstone bridge. This architecture yields fivefold sensitivity enhancement and 25 µm resolution, overcoming LM sensors’ micro‐strain limitations and enabling stable, high‐precision monitoring of multidirectional physiological micro‐motions.

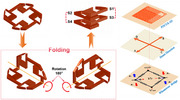

## 1 Introduction

The rapid advancement of soft strain sensors is driving a paradigm shift in cutting‐edge fields such as medical monitoring [1], wearable electronics [2], electronic skin [3, 4], and soft robotics [5, 6, 7]. Conventional commercial strain gauges are typically fabricated by depositing strain‐sensitive metals onto flexible substrates such as Polyimide (PI) and Polyethylene Terephthalate (PET) films [8, 9, 10, 11]. Such flexible strain gauges are widely used in construction and aerospace [12, 13]. However, due to their intrinsically high elastic modulus and extremely low stretchability (<0.1%, essentially non‐elastic), they can be bent or folded but cannot be stretched [1, 12, 14]. This results in a pronounced mechanical mismatch with human skin. Consequently, when attached to soft biological tissues like human skin, the weak tension generated by the skin is insufficient to induce measurable deformation in these conventional sensors, making them unable to directly or accurately reflect the true deformation of soft biological tissues [15, 16]. In contrast, soft strain sensors feature ultra‐low modulus and high stretchability, enabling seamless and conformal adhesion to human skin or robotic surfaces [17, 18, 19, 20, 21, 22]. They can deform synchronously with soft tissues, allowing continuous real‐time tracking and highly sensitive dynamic behavior monitoring [23, 24, 25, 26].

These stretchable strain sensors are typically fabricated by printing or depositing stretchable conductors or semiconductors—such as carbon nanotubes (CNTs) [27], metal nanowires (MNWs) [28], conductive hydrogels [29], and liquid metal (LM) —onto elastomeric substrates to form strain‐sensitive networks, when a soft sensor is stretched, its capacitance, inductance, and resistance undergo predictable changes. The most common type of stretchable strain sensor is the resistive strain sensor. The type of conductive material and the structure of the conductive pathways influence its resistance variation characteristics, thereby affecting the sensor's performance. Despite their high sensitivity [30, 31, 32], carbon‐based strain sensors are often suffer from limited stretchability(usually < 50%)and cannot reliably accommodate large deformations [33]. Similarly, while MNWs exhibit excellent conductivity, their percolated conductive networks are vulnerable to mechanical fatigue, fracture, and incomplete recovery under repeated loading cycles [32]. Hydrogels, while notable for their outstanding biocompatibility and tissue‐like softness, are constrained by low electrical conductivity and environmental instability due to their intrinsically high water content [19]. In contrast, room‐temperature LM (such as gallium‐indium alloys) uniquely combine metallic‐level conductivity with fluidic deformability [2, 24, 34]. Resistance changes in LM channels can be analytically predicted based on the sensor's stretching direction and dimensions, where macroscopic dimensional changes determine a deterministic strain‐resistance relationship [26]. Their ability to withstand extreme mechanical deformation (>500% strain without fracture), combined with intrinsic self‐healing driven by fluidic flow, enables LMs to achieve exceptional mechanical compatibility with soft biological tissues [7, 9, 21, 22, 25, 35]. These unique attributes position liquid metals as ideal candidates for next‐generation stretchable and biocompatible strain sensors.

LM‐based strain sensors can effectively track large‐scale body movements—such as knee and elbow flexion—owing to the exceptional stretchability [17]. However, LM sensors typically exhibit a relatively low gauge factor (GF, usually< 1), which limits their ability to detect subtle physiological signals associated with ultralow deformations [36, 37]. For instance, they prove unable to precisely monitor minute movements of the eye. To address this, enhancing the sensitivity of LM sensors has become a key research focus. Existing strategies primarily fall into two categories. The first involves materials engineering, which utilizes highly sensitive nanomaterials to form composite structures. For example, recent studies have leveraged the synergistic effects of MXene and CNTs to optimize the structure and interfaces of conductive networks through self‐assembly or by integrating MNWs and liquid metals [32], thereby enhancing sensitivity, linearity, and self‐healing capabilities. However, this introduces a fundamental trade‐off: while nanofillers increase the gauge factor (GF), the incorporation of nanoparticles increases the composite's viscosity and stiffness. This prevents the material from maintaining the fluidic behavior inherent to LM under large deformations, thereby sacrificing the sensor's stretchability and conformability [30, 31]. Second, microstructural engineering—such as serpentine layouts or pre‐stressed architectures—offers an alternative approach to enhancing sensitivity [20, 38]. Recent work has demonstrated significant increases in GF and overall sensing performance by incorporating structural gating mechanisms within LM channels [5, 6]. These advances are achieved through approaches such as using heterogeneous elastomers to modulate LM channel contraction and closure [5], or embedding LM‐impregnated foams to dynamically regulate the connectivity of conductive pathways [6]. These designs achieve geometric amplification by translating minute material strains into larger structural deformations. However, while conceptually appealing, these designs face significant fabrication challenges in practical implementation. Such intricate microstructures typically rely on complex, high‐precision, multi‐step manufacturing processes like photolithography, etching, or fine‐pitch mold casting. These techniques are not only costly but also demanding on equipment and environmental conditions, resulting in low production yields. Consequently, there is an urgent demand for stretchable sensors that circumvent these trade‐offs and simultaneously offer high sensitivity, large strain range, and low cost.

Here, we propose FOCUS—a four‐in‐one consolidated unison strain sensor specifically designed for monitoring ultra‐low strain with enhanced sensitivity. A straightforward yet efficient fabrication strategy is adopted, in which a 2D planar structure is seamlessly transformed into a unique 3D orthogonal structure via planar screen‐printing and folding. The LM sensor, a key component of FOCUS, exhibits predictable strain‐induced resistance changes in different directions [7, 26]. Its resistance increases under vertical stretching and decreases under horizontal stretching [26]. Leveraging this characteristic, we integrated four LM sensors into a single unit and configured them in a full‐bridge Wheatstone circuit to create FOCUS. This design achieves an innovative sensitivity enhancement exceeding fivefold (Table S1). Benefiting from this design, FOCUS enables cross‐scale strain detection ranging from centimeters down to microns with an ultrafine resolution of 25 µm, while providing full‐axial strain monitoring, excellent cyclic stability (>5000 cycles), and high resistance to thermal drift (25°C–75°C). We further validated the reliable performance of FOCUS in periocular micromotion tracking and subtle human joint deformation monitoring, offering a promising tool for clinical assistive diagnostics. We believe that FOCUS, empowered by its innovative 3D orthogonal architecture, will open new paradigms in wearable electronics, soft robotics, and precision medicine. Compared to other soft strain sensors (Table S2), FOCUS demonstrates superior overall performance. It achieves significantly enhanced sensitivity and full‐axial strain detection without compromising the inherent flexibility and ultra‐high tensile properties (Stretching approaches 1000%) of the material. Fabrication is accomplished using ultra‐simple, low‐cost screen printing and folding step.

## 2 Results and Discussion

### 2.1 The Structure and Sensitivity Amplification Mechanism of the FOCUS

**FIGURE 1:**
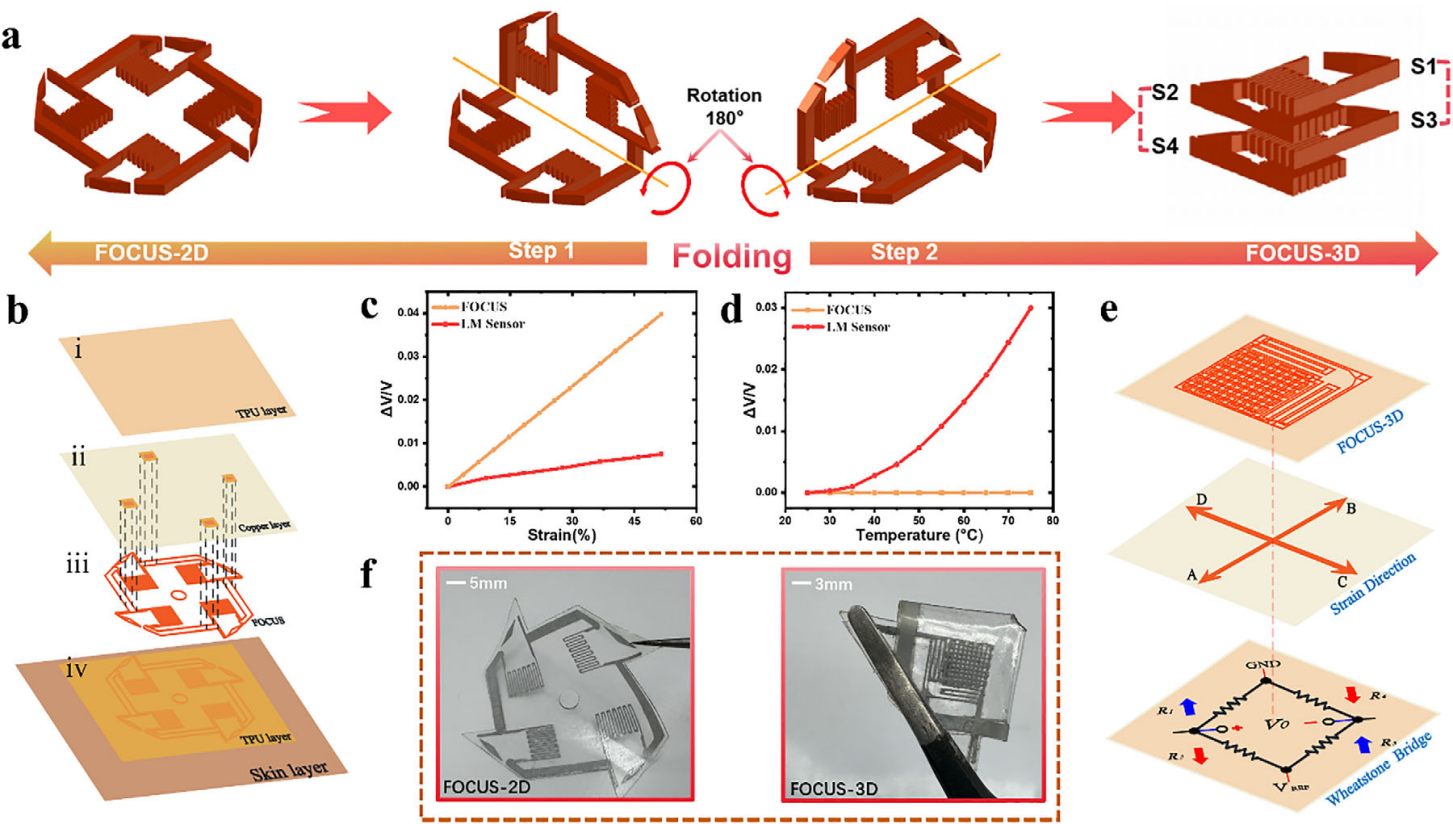
Schematic of FOCUS folding and Optical Schematic Diagram. (a) Illustration of FOCUS conversion from 2D to 3D, the core process involves a two‐step folding procedure. (b) Exploded diagram of the FOCUS‐2D. (c) Comparison chart of FOCUS and LM sensor sensitivity performance. (d) Comparison chart of FOCUS and LM sensor temperature resistance performance. (e) Schematic diagram of FOCUS‐3D spatial configuration, illustrating the strain direction of FOCUS. (f) Optical images of FOCUS in 2D and 3D state.

The core of the FOCUS structure lies in its unique four‐in‐one consolidated structure and the resulting sensitivity amplification mechanism. This is closely related to the tensile principle of LM sensors and the differential principle of the Wheatstone bridge. The FOCUS comprises four identical LM sensor units S1, S2, S3 and S4, (Figure S1), the resistance values of these four LM sensors are identical, each measuring 6 mm × 8 mm. We transformed FOCUS‐2D into a unique 3D orthogonal structure through a two‐step 180‐degree folding process along the symmetry axis (Figure 1a). Following hot pressing, FOCUS‐3D was integrated into a monolithic structure. This design ingeniously leverages the opposing strain effects of LM sensors under biaxial stretching, thereby achieving a full‐bridge sensitivity enhancement.

Specifically, during FOCUS stretching, the resistance changes of S1 and S3 increase, yielding positive values; the resistance changes of S2 and S4 decrease, yielding negative values [17, 26]. Therefore, in the voltage output formula of the Wheatstone bridge [20].

$$\;V_{\mathrm{out}}=V_{\mathrm{in}}\;.\frac{1}{4}\left(\frac{\Delta R_{S1}}{R_{0}}-\frac{\Delta R_{S2}}{R_{0}}+\frac{\Delta R_{S3}}{R_{0}}-\frac{\Delta R_{S4}}{R_{0}}\right)$$

The output voltage is positively superimposed in the calculations of the four sensors, collectively enhancing sensitivity to achieve the maximum sensitivity of FOCUS. Compared to a single LM sensor, it achieves the highest sensitivity.

The FOCUS sensor in a 2D plane consists of four layers: an upper TPU layer, copper, and FOCUS, lower TPU layer (Figure 1b). Diagonally positioned sensors (S1‐S3 and S2‐S4) become spatially parallel after folding, while adjacent sensors align perpendicular to each other, forming a unique 3D orthogonal structure (Figure S2). Following hot pressing, FOCUS‐3D integrates into a consolidated structure. At this point, under FOCUS stretching, the resistances of the two sets of diagonal arms change in opposite directions (Figure S1). Specifically, the resistances of S1and S3 increase in tandem, while those of S2 and S4 decrease in tandem. The differential output characteristic of the bridge ensures that the signal contributions from all four arms are superimposed in the same direction. It's normalized output voltage. Compared to LM sensor, the FOCUS exhibits the greatest improvement in sensitivity (Table S1). Through differential configuration, S2 and S4 are inversely superimposed, contributing to the positive voltage output. The differential operation of the bridge effectively adds the minute signals generated by all four arms in phase, maximizing the enhancement of sensor sensitivity.

FOCUS achieves a breakthrough with sensitivity exceeding five times that of LM sensor (Figure 1c). Simultaneously, it operates reliably from room temperature to high temperatures without exhibiting temperature drift, owing to the intrinsic properties of the Wheatstone bridge, FOCUS exhibits a stable horizontal line (Figure 1d). It can deliver stable output across a wide temperature range from room temperature to 75°C, with minimal impact from temperature fluctuations. This precise and ingenious signal amplification mechanism ensures that the signal contributions from all four LM sensors are amplified in the same direction and fully amplified. In the differential mode of the Wheatstone bridge, the output voltage is the sum of the voltage differences across the two diagonal bridge arms. Therefore, even when each individual LM sensor unit experiences only a minute resistance change, the signals from all four LM sensors are effectively superimposed and amplified, leading to a substantial enhancement of the final output signal. This synergy between the 3D orthogonal structure and the Wheatstone bridge enables FOCUS to efficiently convert subtle external strain into a highly amplified electrical signal, thereby achieving unprecedented ultra‐high sensitivity. The high sensitivity of FOCUS stems from this 2D‐to‐3D structural innovation, the simple folding step enables spatial alignment among the four LM sensors. Through hot pressing, complete interlocking between layers ensures that FOCUS can stretch and function in concert.

The 2D spatial schematic diagram of FOCUS is decomposed into four layers, consisting of four LM sensors arranged on a plane (Figure 1b). In fact, FOCUS resembles the shape of a windmill. We incorporated the windmill's central symmetry into FOCUS, which forms a 3D orthogonal configuration after two folds. Simultaneously, the sensor area is reduced to one‐quarter of its original size. The overall architecture of FOCUS is described in detail as follows (Figure 1b): (i) A TPU layer serves as the top substrate for encapsulating FOCUS; (ii) Based on the Wheatstone bridge principle diagram, four copper plates are distributed at the same positions as the LM connection arms of the four sensors. One set of diagonal copper plates is externally connected to GND and V_REF_, while the other set is externally connected to the positive and negative terminals of a 5V voltage; (iii) Four LM sensors are arranged in a spatially symmetric structure within the plane; (iv) The four sensors are printed onto the bottom TPU film using screen printing technology. The FOCUS‐3D structure is formed by folding the aforementioned 2D plane. After folding, we use hot pressing to integrate the four LM sensors at the same position into a single unit. Spatially, regardless of the folding angle, the final spatial arrangement from top to bottom is always S1, S2, S3, S4(Figure S2). FOCUS‐3D is obtained after folding and hot pressing (Figure 1e), with its dimensions reduced by 75% compared to FOCUS‐2D. The 2D plane and 3D morphology of FOCUS are clearly presented in optical images (Figure 1f).

### 2.2 The Fabrication of the FOCUS and LM Sensor

**FIGURE 2:**
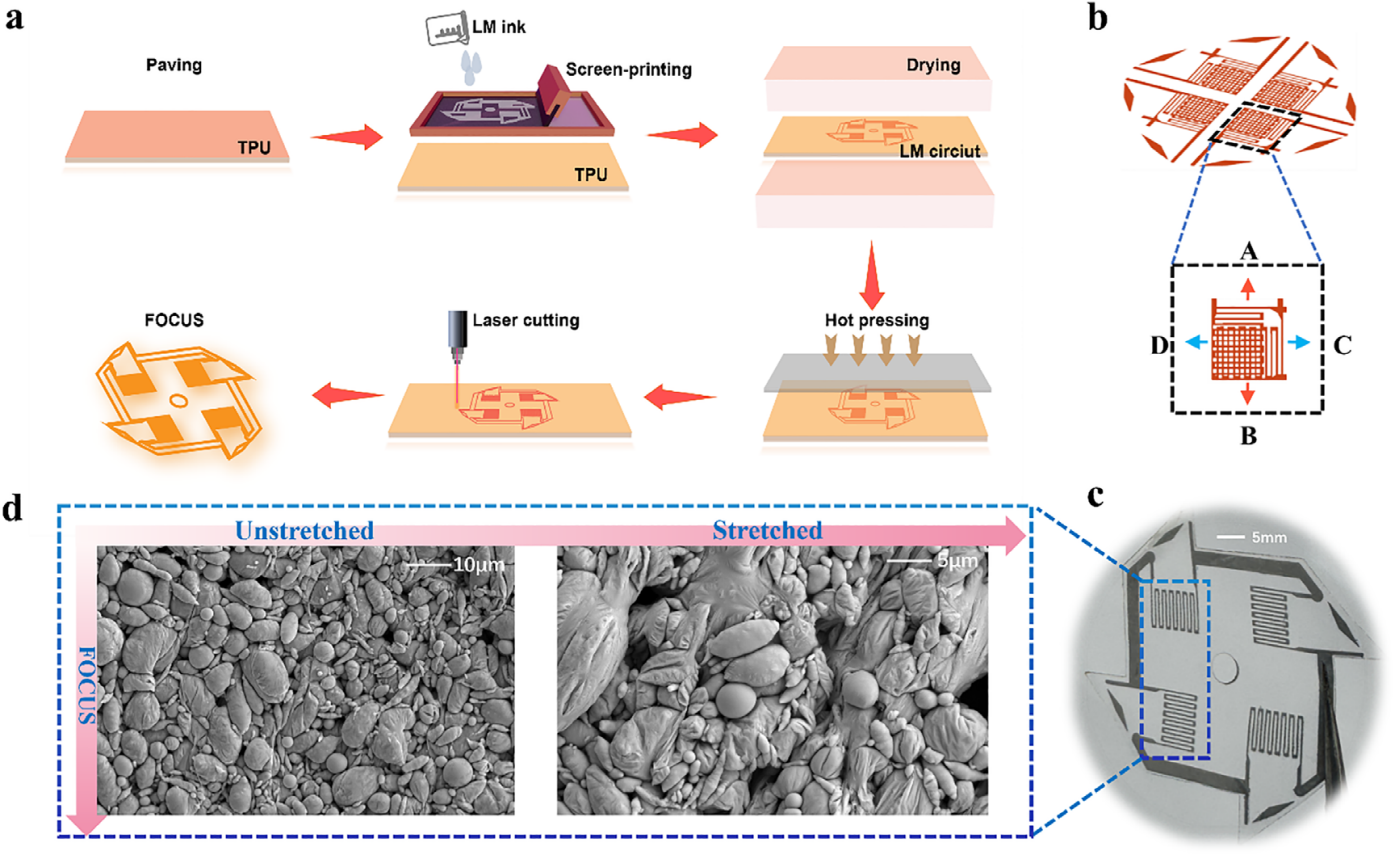
Schematic diagram of the FOCUS fabrication and SEM image. (a) Schematic flowchart of FOCUS preparation. (b) Schematic of full‐axis stretching for FOCUS‐3D. (c) Optical detail image of FOCUS. (d) SEM optical image of FOCUS.

The manufacturing process of FOCUS is a multi‐step procedure, beginning with screen printing LM ink on a TPU membrane (Figure 2a). The LM ink was prepared by sonicating LM in the PVP n‐decanol solution [22]. After heating and drying the patterned TPU film, hot pressing, and laser cutting, the planar structure of FOCUS, designated as FOCUS‐2D, was obtained. In FOCUS‐2D, four identical LM sensors are integrated within the same planar substrate. The FOCUS‐2D was then transformed into the final 3D configuration, FOCUS‐3D, by folding twice along the pattern's axis of symmetry (Figure 1a) and subsequently hot pressing. After forming the FOCUS‐3D through hot pressing (Figure 1e), it achieves full axis strain in all four axial directions A, B, C, and D (Figure 2b).

Simultaneously, we captured scanning electron microscope (SEM) images of the FOCUS sensor. The LM sensor position of the FOCUS‐2D optical image serves as the SEM sampling position (Figure 2c). In the unstretched condition, LM particles remain relatively independent, maintaining their spherical, smooth morphology and intact oxide layer (Figure 2d). In the stretched condition, nearly all large particles (>2 µm) within FOCUS rupture, releasing liquid metal that coalesces into conductive pathways (Figure 2d). When FOCUS is stretched to 50%, termed the activated state, LM particles within FOCUS fracture, completing sintering to form conductive pathways (Figure S3). Therefore, we have attached photographs of the LM sensor fixed to the stage after 50% stretching, along with SEM images of the sensor in its 50% stretched state. As shown in Figure R3, under tensile stress, the liquid metal particles within the LM sensor undergo sintering and fusion, demonstrating exceptional electrical conductivity. We present detailed SEM images at various scales: 1 mm, 100, 50, 30, 20, and 10 µm. All images clearly show the real‐time state of liquid metal particles within a single pathway of the LM sensor. Specifically, the 1 mm SEM image presents a macro view of the liquid metal pathway. From 100 to 10 µm images, LM particles sinter together to form conductive pathways when subjected to 50% strain. Finally, in the stretched condition, nearly all large particles (>2 µm) within FOCUS rupture, releasing liquid metal that coalesces into conductive pathways (Figure 2d).

### 2.3 Characterization of the LM Sensor

FOCUS is composed of four identical LM sensors, making the stretching behavior of LM a critical determinant of performance, since LM sensors exhibit anisotropic responses when stretched along different directions. We define the LM sensor as initial condition; direction a stretching; direction b stretching, each displaying a physical diagram of the stretching process. Here, we define the liquid metal channel width in the LM sensor as *W*, the axial stretching channel length as *L*, and the cross‐sectional height as *d*. Stretching in direction a and direction b produces different strains on the LM sensor (Figure S4), It clearly demonstrates the three states of the lunar module sensor: initial state (unstretched); stretched in direction a; stretched in direction b (Figure S4). The resistance R depends on the resistivity ρ of the liquid metal LM path, the effective length L, and the effective cross‐sectional area A: $R=\rho \frac{L}{A}$[22].

When stretched along direction a, the LM sensor's resistance change rate continues to rise until strain approaches 1000% (Figure S5). When stretched in direction b, resistance primarily decreases within the 50% strain range, then increases with continued stretching. This indicates that the strain effect of the LM sensor differs depending on the direction of stretching. As per the voltage output formula of the Wheatstone bridge, it precisely exploits the fact that within the 50% strain range, the strain effects in directions a and b are opposite. By applying differential summation, this generates a fully positive output.

**FIGURE 3:**
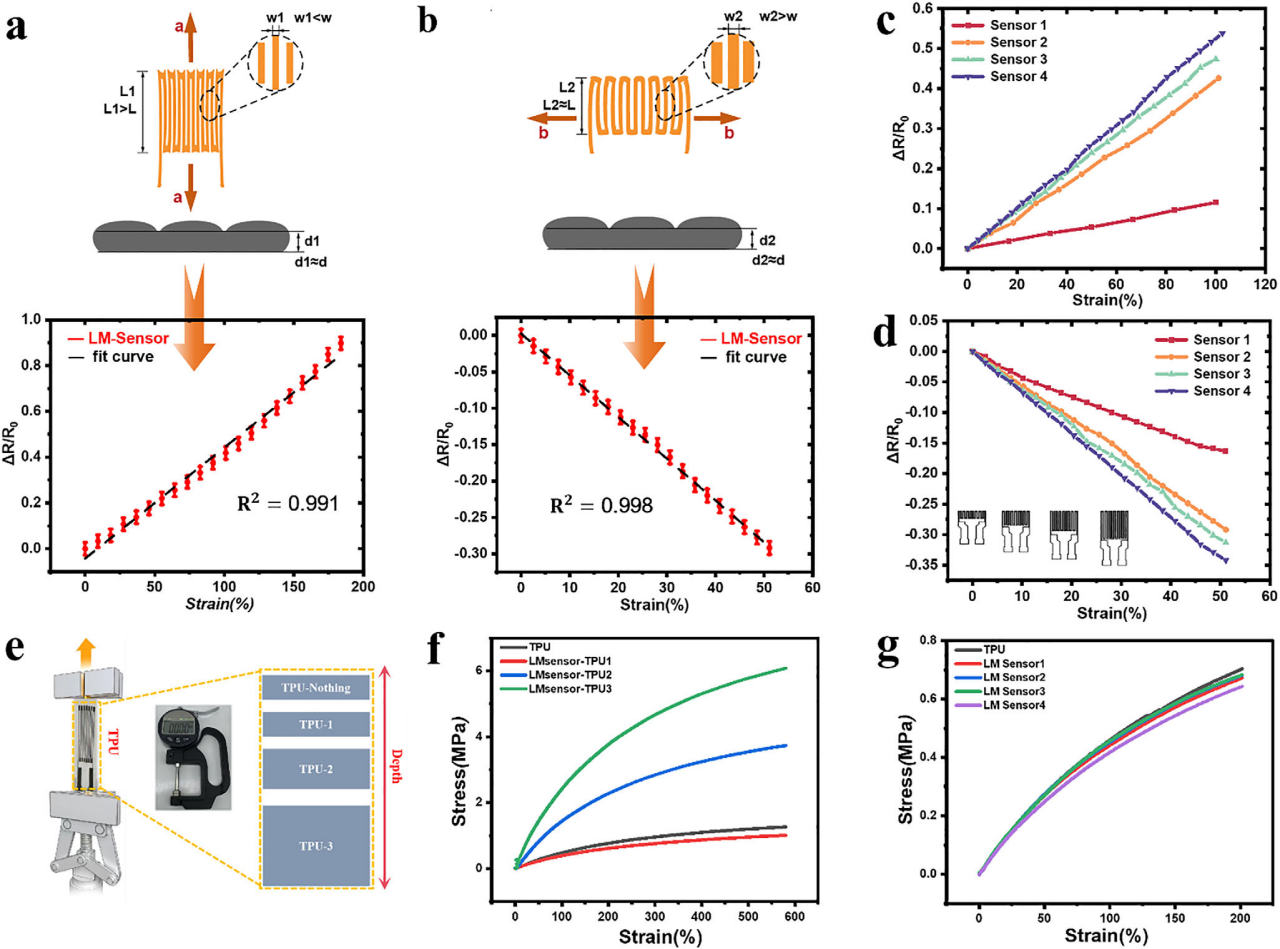
Characterization of LM Sensor. (a) Schematic diagram of LM sensor stretching along direction a and sensitivity curve when stretched over 150%. (b) Schematic diagram of LM sensor stretching along direction b and sensitivity curve when stretched over 50%. (c) Comparison of sensitivity curves of LM sensors of different sizes when stretched along the direction a by 100%. (d) Comparison of sensitivity curves of LM sensors of different sizes when stretched along the direction b by 50%. (e) Schematic Diagram of Sensor Stretching on Substrates of Different Thicknesses. (f)Stress comparison diagram of LM sensors on substrates with different thicknesses. (g)Stress comparison diagram of LM sensors with the same thickness but different dimensions.

We magnified the sensitivity curves of the LM sensor stretched 150% in the direction a (Figure 3a) and 50% in the direction b (Figure 3b), accompanied by an intuitive schematic of the LM sensor's stretching mechanism. To meet the requirements for micro‐scale strain measurement, we employed the stretching principle of the LM sensor within the 50% strain range to construct FOCUS. Thus, the sensitivity amplification mechanism of FOCUS is achieved through an innovative combination of the LM sensor strain principle and the Wheatstone bridge differential mechanism (Figure S6).

We conducted electrical characterization of LM sensors under direction a and b stretching. When the LM sensor deforms under mechanical tension, the resistance of the sensor changes. By measuring the specific value of the resistance change, the applied strain can be calculated and confirmed (Figure S5). The resistance change of the sensor is directly proportional to the applied stress. Its sensitivity curve amplified within the 150% strain range, where GF reaches 0.4 at 100% stretch (Figure 3a). When stretched along the direction b, the sensor signal first decreases as strain increasing, then reverses as strain continues to increase (Figure S5). The sensitivity curve of the LM sensor in the direction b clearly shows a decreasing trend in sensitivity within the 50% strain range (Figure 3b). At this point, the sensor's strain sensitivity exhibits monotonicity, with sensitivity decreasing as strain increases, and the absolute value of GF is 0.6. After fitting, the R‐squared values of the LM sensor's sensitivity curves are 0.991 and 0.998(Figure S5), respectively, both demonstrating excellent linear relationships. The LM sensor demonstrates good extensibility under stretching, from the initial stretched state to the natural stretched state in both the a and direction bs (Figure S7). However, to monitor micro‐strains associated with periorbital or joint micro‐movements, sensors with higher sensitivity are required to capture subtle physiological signals. We aimed to develop FOCUS to address this limitation (Figure S6).

Beyond the electrical characterization of LM sensors, we further investigated the influence of channel length [21, 22] and substrate thickness on their sensitivity. Subsequently, the ratio of the sensor's Y‐axis axial length dimensions is also one of the factors affecting GF. Therefore, we prepared four sensors of different sizes. We conducted experiments to investigate the effect of sensor longitudinal length on sensitivity. In the experiments, only the longitudinal length of the sensor was altered. Using the longitudinal length L of the LM sensor as the reference, we prepared a set of sensors of varying lengths, arranged top to bottom as follows: Sensor 1 (3 mm); Sensor 2 (6 mm, LM sensor); Sensor 3 (8 mm); and Sensor 4 (12 mm) (Figure S8). The electric track was then set to the same speed and subjected to uniform axial stretching. The comparison of sensitivity curves for the four sensors showed that Sensor A had the lowest sensitivity, while Sensor D had the highest sensitivity (Figure 3c). Although the sensitivity differences between Sensors 2, 3, and 4 were small, sensitivity increased with an increase in the sensor's Y‐axis length. Additionally, compared to the other three sensors, Sensor 1 had lower sensitivity. In LM sensors, sensitivity increases with the increase in sensor longitudinal length. This also applies to sensor transverse stretching. This indicates that within the stretching range of LM sensors (50% strain discussed in this experiment), the longitudinal length of LM sensors is positively correlated with their sensitivity. During stretching in the direction b, the experimental results were largely consistent with those in the direction a. Sensor 1 exhibited the slowest rate of resistance decrease, while the other three sensors showed a relatively concentrated trend (Figure 3d).

For mechanical characterization, we investigated the influence of the substrate (TPU) thickness on the sensitivity of the encapsulated LM sensor (Figure 3e). We fabricated LM sensors encapsulated in TPU substrates of varying thicknesses, designated as TPU‐1, TPU‐2, and TPU‐3, with corresponding numerical thickness increments (Figure S9). The sensitivity of the LM sensor decreases as the substrate thickness increases. This is because a thicker TPU substrate enhances the overall stiffness of the sensor (Figure 3f). While the LM component itself is very soft, the thicker TPU most of the strain during stretching, which reduces the effective strain transferred to the LM conductor and leads to a less pronounced change in its resistance. This indicates that an increase in substrate thickness limits the deformation capability of the LM channel, reducing strain response efficiency and thereby hindering the realization of high‐sensitivity sensors [17].

After successfully determining the effect of substrate thickness on sensitivity, we proceeded to investigate another important design variable, sensor dimensions, for a systematic mechanical characterization of the sensor's performance. By keeping the substrate thickness constant, we were able to precisely evaluate the independent influence of sensor dimensions on device sensitivity and overall mechanical response. Additionally, we conducted tensile tests on LM sensors with different dimensions but consistent TPU thickness to further assess the impact of size on mechanical properties. The LM sensor operates within 200% strain range, the stress‐strain curve slopes of LM sensors with different dimensions were nearly identical, indicating that their mechanical response is independent of size (Figure 3g). This suggests that, due to the high flexibility of our LM sensors at the micrometer scale, strain can be effectively transmitted and uniformly distributed during force‐induced deformation. Therefore, within a certain range, the size of LM sensor has a negligible impact on their overall mechanical response.

### 2.4 Characterization of the FOCUS

FOCUS‐3D consists of four LM sensors arranged perpendicular to each other in a 3D orthogonal structure. When FOCUS is stretched, S1 and S3 stretch in the direction a, resulting in increased sensitivity, while S2 and S4 stretch in the direction b, causing a decrease in sensitivity within a 50% strain range. By differentially summing the resistance changes across four sensors via a Wheatstone bridge, the four LM sensors operate in coordinated synchronization within the sensor's 50% strain range when FOCUS is stretched. This design constitutes the core of the four‐in‐one configuration, enabling FOCUS to achieve an amplification factor exceeding five times that of a single LM sensor. A more notable feature of FOCUS is that the A, B, C, and D directions represent the four axes of the Cartesian coordinate system, achieving consistent stretchability along all axes and enabling strain monitoring in all directions (Figure 2b).

**FIGURE 4:**
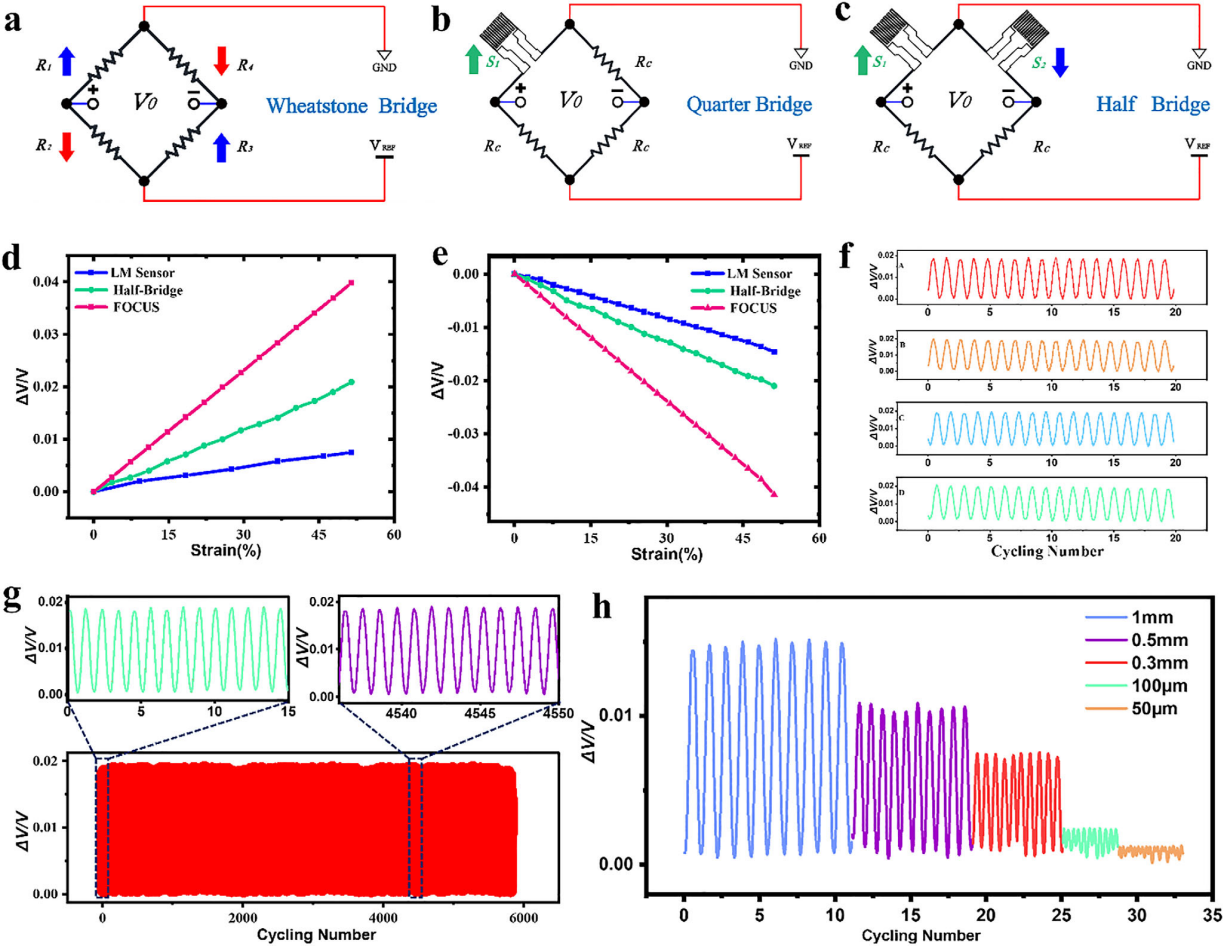
Electrical and mechanical characterization of FOCUS. (a)Wheatstone bridge diagram. (b) Schematic diagram of single‐bridge sensor bridge connection. (c) Schematic diagram of half‐bridge sensor bridge connection. (d) Sensitivity comparison of three bridge‐arm sensors under tensile loading in direction a. (e) Sensitivity comparison of three bridge‐arm sensors under tensile loading in direction b. (f) Comparison of full axial tensile cycling. (g) FOCUS exceeds 5000 cycle test chart. Two cyclic segments extracted. (h) FOCUS‐3D cyclic comparison at 1, 0.5, 0.3, 100, and 50 µm.

In fact, the three different sensor configurations involve varying numbers of sensors connected to the Wheatstone bridge (Figure 4a). We verified the sensitivity of FOCUS‐3D through comparative experiments. To clearly demonstrate the significant sensitivity improvement of FOCUS compared to LM sensors and the quantitative relationship between them, we configured a quarter‐bridge sensor (Figure 4b) and a half‐bridge (Figure 4c). The quarter‐bridge sensor configuration represents testing of a single LM sensor, providing a direct hot pressing for sensitivity order‐of‐magnitude comparisons with FOCUS sensors. Subsequently, we fabricated half‐bridge sensors that directly amplified sensitivity by coupling two LM sensors. The half‐bridge test not only validated the principle of signal amplification but also served as a crucial bridge connecting the performance of single‐bridge sensors to that of the FOCUS. By systematically comparing these three configurations, we clearly demonstrated a stepwise performance enhancement from the LM sensor to FOCUS architecture, powerfully validating the superiority of the FOCUS design in achieving ultra‐high sensitivity. By preparing these three types of sensors, we conducted experiments to compare their sensitivity curves under the same strain conditions and validate the performance improvement of FOCUS. FOCUS full bridge connects to the Wheatstone bridge, the quarter bridge connects to S1 (Figure S10) and the half bridge connects to S1 and S2 (Figure S11). Therefore, following the above method, FOCUS‐3D, half‐bridge, and single LM sensor were fabricated. The GF values for the three sensor configurations were as follows: along the direction a, LM sensor: half‐bridge sensor: FOCUS‐3D = 2:5:10 (Figure 4d). Along the direction b, LM sensor: half‐bridge sensor: FOCUS‐3D = 3:5:10 (Figure 4e). Consequently, the FOCUS‐3D exhibited a fivefold improvement in sensitivity compared to the LM sensor. These three type sensors were subjected to uniform strain along the a and b axes on an electrical rail, generating voltage‐strain curves, or sensitivity images. All images clearly demonstrate the significant improvement in FOCUS sensitivity. The sensitivity curves for the FOCUS‐3D, half‐bridge, and LM sensor along the direction a are compared (Figure S12). The sensitivity curves for the FOCUS‐3D, half‐bridge, and quarter‐bridge sensors along the direction b are compared (Figure S12). This means that FOCUS excels in both sensitivity and directionality, and its sensitivity can be freely adjusted by controlling the LM sensor size. Furthermore, the FOCUS‐3D has essentially the same size as a quarter‐bridge sensor, measuring 8 mm × 8 mm.

FOCUS's stability, response speed, and durability are also important indicators for evaluating its performance. We loaded the FOCUS‐3D to 25% strain and then unloaded it to zero strain at a rate of 1 mm/s while measuring its potential change. Simultaneously, we conducted tensile cycle tests on axes A, B, C, and D, with nearly identical strain capacities across all axes. We selected 20 cycles from these tests for demonstration (Figure 4f). To further validate the durability of FOCUS, which was stretched to a 1 mm stretching distance more than 5000 cycles, and its potential change was monitored. Despite the increasing number of stretch‐release cycles, the test curve remained highly stable (Figure 4g). Recent research on flexible sensors, particularly in the field of flexible strain sensors, typically presents durability cycle test results ranging from approximately 500 to 2000 cycles [21, 39, 40, 41, 42]. This is a common and sufficient standard for validating the mechanical fatigue performance of sensors. At FOCUS, we have conducted over 5000 tests to achieve a high standard in the durability verification of flexible sensors. The inset shows details of the first and middle 10 cycles. Furthermore, the cyclic performance of the FOCUS‐3D sensor at five different strain levels: 1, 0.5, and 0.3 mm; 100 and 50 µm (Figure 4h). FOCUS demonstrated excellent stability and repeatability. To further investigate whether FOCUS responds at smaller strains, we conducted a gradient strain experiment at 200 mm/s: FOCUS underwent tensile and release cycles at 25 µm intervals (less than 0.5% strain), followed by release at the same gradient intervals after reaching 5% strain (Figure S13). Ultimately, FOCUS exhibited more micro resolution, with its GF within 5% strain overlapping relatively closely with the sensitivity curve over the 50% strain range. This indicates that FOCUS possesses a resolution of approximately 25 µm and maintains excellent linearity within 5% strain (Figure S13). Meanwhile, we applied a 5% strain at a speed of 200 mm/s. The FOCUS demonstrated an excellent response time of 56 ms, with a recovery time of 52 ms after the response (Figure S14). Based on the above characterization, this also means that FOCUS, with its unique 3D orthogonal structure, can detect strain in the A, B, C, and D directions with exceptional directional sensitivity, offering full‐axis strain capability across the plane, arriving 25 µm resolution.

**FIGURE 5:**
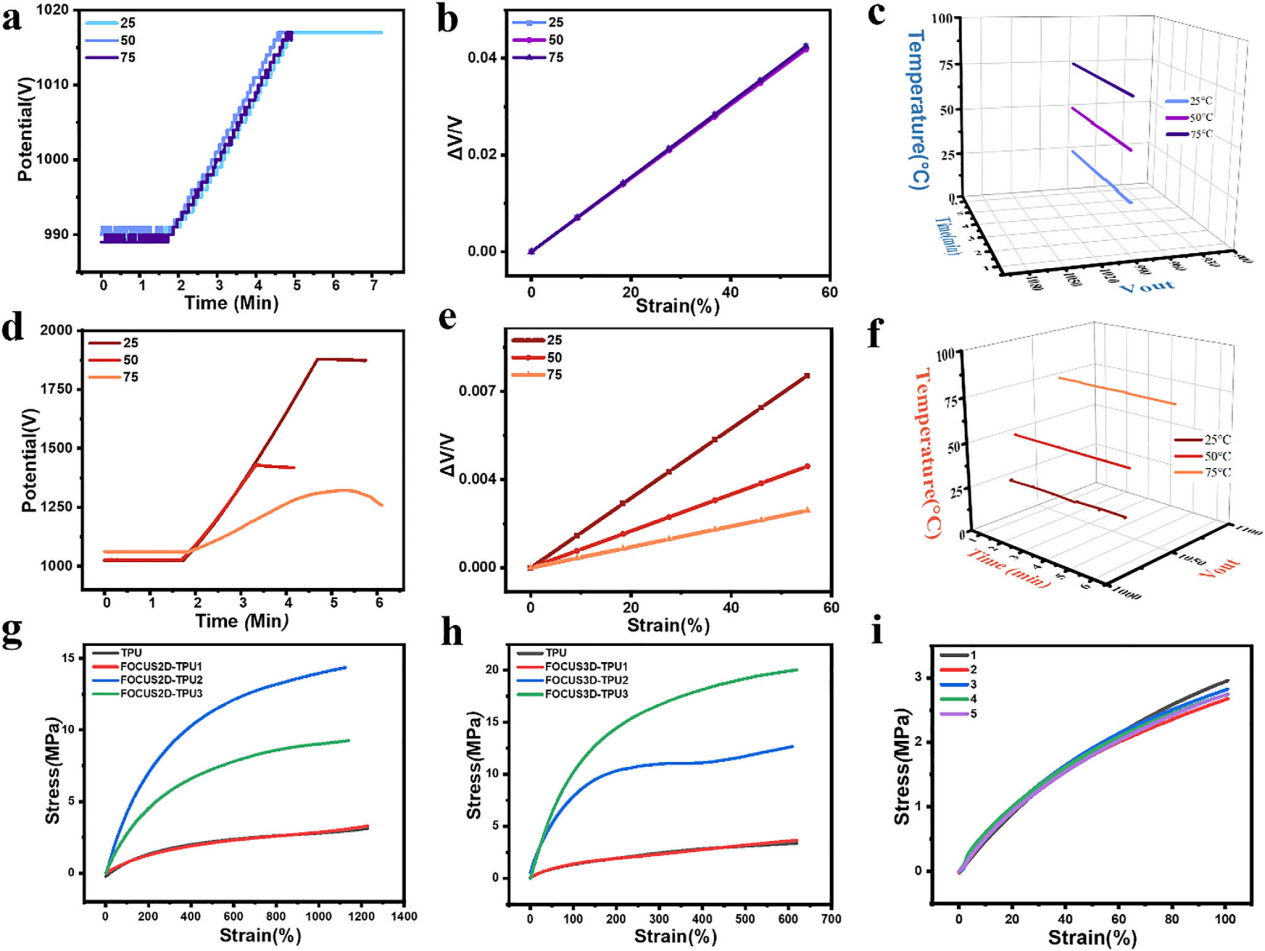
FOCUS‐3D tensile performance test diagram. (a) FOCUS‐3D strain potential variation diagram at different temperatures when stretching. (b) FOCUS‐3D sensitivity comparison diagram at different temperatures. (c) FOCUS‐3D static waterfall plot for three metrics: temperature, potential, and time. (d) LM Sensor strain potential variation diagram at different temperatures. (e) LM Sensor sensitivity comparison diagram at different temperatures. (f) LM sensor static waterfall plot for three metrics: temperature, potential, and time. (g) Stress Comparison Diagram for Three Different Thickness Substrates: Empty TPU and FOCUS‐2D.(h) Stress Comparison Diagram for Three Different Thickness Substrates: Empty TPU and FOCUS‐3D.(i) FOCUS‐3D Five‐Stress Comparison Diagram.

Subsequently, to test FOCUS ability to withstand temperature fluctuations, we tested and compared it at three temperatures: room temperature, 50°C, and 75°C (the sensor was heated in an oven for 5 min). First, we tested FOCUS's performance under dynamic stretching. During stretching, the slopes of the FOCUS‐3D's potential curves at the three different temperatures were essentially the same, demonstrating excellent stability (Figure 5a). FOCUS's sensitivity remained essentially consistent at the three different temperatures during stretching (Figure 5b). Under static, unstretched conditions, the potential values at the three different temperatures remained essentially flat, showing no drift, demonstrating exceptional stability (Figure 5c). This verifies that under no‐stretch conditions, despite continuously heating the FOCUS sensor on a heating stage, its output potential remains perfectly parallel—meaning no drift, demonstrating excellent static stability. To evaluate the dynamic tensile properties of FOCUS under temperature cycling conditions, we conducted dynamic temperature‐cycle strain experiments (Figure S15). Throughout both the temperature rise and fall phases, FOCUS exhibited stable sensitivity with no drift observed. We subjected the material to a temperature cycle from 0°C to 75°C, applying a 6% tensile strain at each corresponding temperature interval. Subsequently, at 42% strain, we released the material to 0% strain using a 6% gradient. Throughout the cyclic tensile test, sensitivity remained linear and was virtually unaffected by temperature.

Due to the inherent resistance to temperature drift provided by the combination of the LM sensors and the Wheatstone bridge, the performance of FOCUS remains stable in extreme environments, whether at room temperature or high temperatures. For comparison, we measured the voltage output curves of individual LM sensors during dynamic uniform stretching after heating them to various temperatures in an oven (Figure 5d). As temperature increases, the sensor's sensitivity exhibits a marked decrease (Figure 5e). Subsequently, we plotted their sensitivity curves at different temperatures, revealing a decrease in sensitivity with increasing temperature. Finally, by generating a 3D waterfall plot of temperature, stretching time, and voltage output (Figure 5f), we clearly demonstrated that the baseline voltage of the LM sensor shows a significant upward drift with rising temperature. This proves that temperature induces drift in the LM sensor under both static and dynamic strain, standing in stark contrast to the stable state of the FOCUS sensor in its corresponding 3D plot (Figure 5c). This demonstrates the excellent temperature self‐compensation capability of FOCUS, which addresses the high‐temperature drift issue of the LM sensor. Therefore, FOCUS exhibits excellent stability and resistance to temperature drift under both dynamic and Wide temperature tolerance range. Its resistance to temperature drift relies primarily on the differential measurement principle and the common‐mode rejection capability of the bridge circuit. Since all four sensing units employ identical LM material and are exposed to identical thermal conditions, resistance drift induced by ambient temperature variations occurs synchronously and symmetrically across all four resistive arms (Figure 4a). This drift is maximally suppressed and eliminated by the differential measurement mechanism of the bridge circuit.

Finally, the mechanical properties of FOCUS were characterized. We tested the mechanical properties of FOCUS in both 2D and 3D states respectively. Compared to a bare TPU film of the same size and thickness, the stress‐strain curves of FOCUS‐2D were almost identical up to a strain of 130%, indicating that FOCUS‐2D had minimal impact on the mechanical properties (Figure 5g). In both experiments with increasing TPU thickness, FOCUS‐2D required greater force to stretch, meaning greater stress was required at the same strain. Therefore, substrate thickness is a factor influencing the stretchability of LM sensors. In the mechanical properties experiments of FOCUS‐3D (Figure 5h), the thicker the substrate, the greater the stress required, which is consistent with the conclusions from experiments with different FOCUS‐2D thicknesses (Figure 5g). After five cycles of stress stretching, the stress‐strain curves of FOCUS‐3D essentially overlapped, demonstrating excellent recoverability and highly stable (Figure 5i). In general, the experiments strongly demonstrated that the FOCUS sensor maintains the original softness of TPU in both 2D and 3D states, its overall mechanical properties can be effectively regulated by the thickness of TPU, and it still maintains excellent cyclic stability and recoverability in the 3D structure.

### 2.5 Application of FOCUS as a Wearable Sensor

The designed and fabricated FOCUS sensor, with its high sensitivity, excellent stability, and good biocompatibility, is broadly applicable for monitoring human skin and joint deformation. FOCUS, due to its miniature size and exceptional sensitivity, is specifically targeted at the periorbital region—particularly key strain concentration points around the eyes—converting minute eye movements into measurable skin‐level deformations (Figure S16). Ocular motility serves not only as a core component of the visual system but also as a sensitive indicator reflecting the state of the central nervous system. Precise, real‐time monitoring of eye movements holds significant value for clinical diagnosis and emerging applications. Eye movements such as saccades and fixation are complex processes precisely regulated by the brain. Consequently, they can to some extent reflect diseases or abnormalities within the human body, holding immense clinical diagnostic significance. Future devices like flexible sensors that adhere to the skin will serve as non‐invasive precision tools, providing critical assessment methods for disease diagnosis and cognitive impairment. Beyond clinical applications, FOCUS will also become an essential component in research and monitoring areas such as human‐computer interaction, fatigue and cognitive load detection, and attention and learning assessment.

**FIGURE 6:**
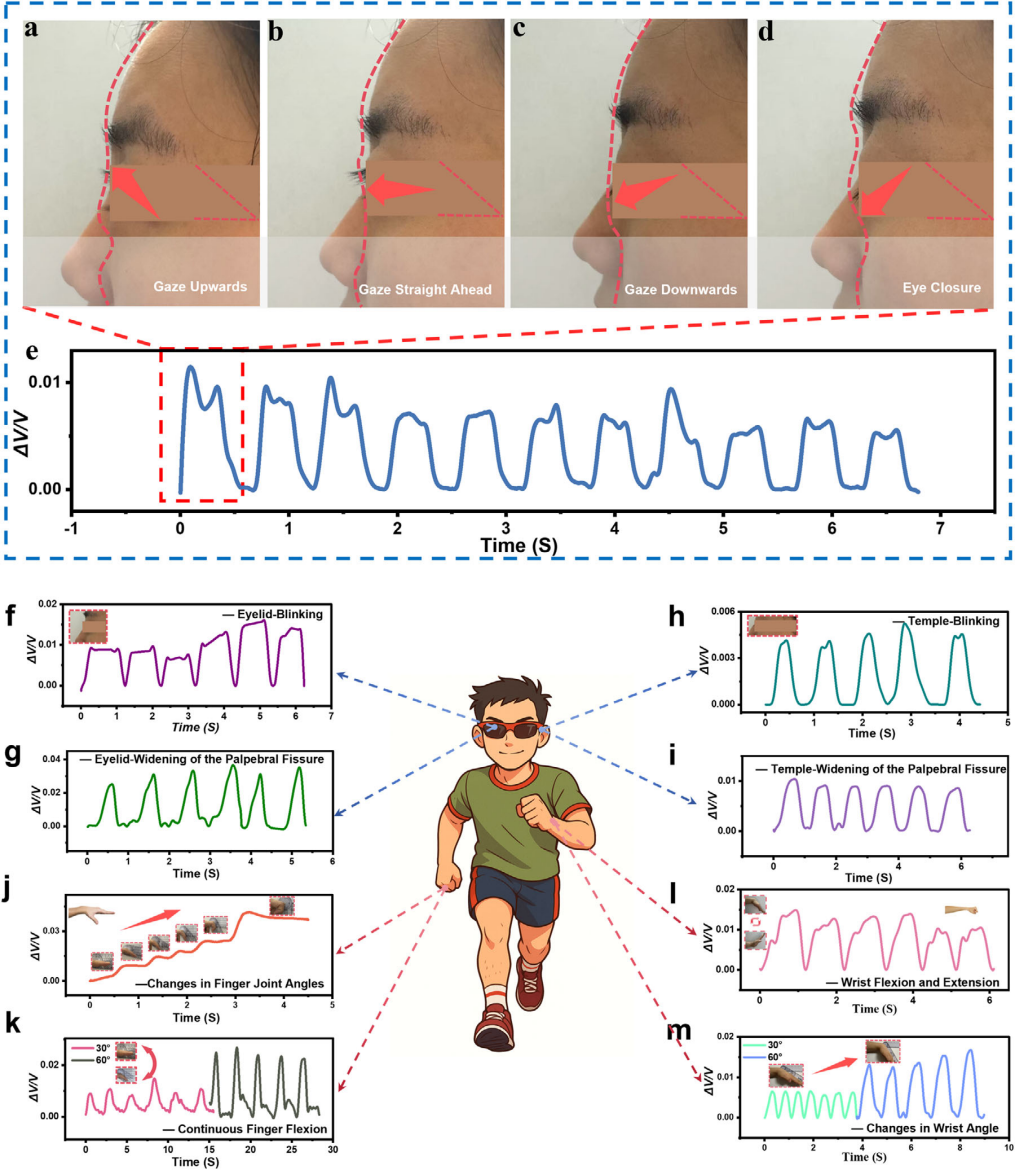
Vertical Scanning Movement 3D Face Scan and FOCUS application physiological signal diagram. (a) Gaze Upward. (b) Gaze Straight Ahead. (c) Gaze Downward. (d) Eye Closure. (e) Vertical saccade task Signal Acquisition Diagram. (f) Schematic diagram of upper eyelid free blink signal acquisition. (g) Schematic diagram of upper eyelid open‐eye signal acquisition. (h) Schematic diagram of blink signal acquisition at the temple location. (i) Schematic diagram of eye opening signal acquisition at the temple location. (j) Finger Joint Angle Variation Motion Signal Acquisition Diagram. (k) Schematic diagram of signal acquisition for continuous finger flexion at 30° and 60°. (l) Wrist Joint Free Up‐Down Movement Signal Acquisition Diagram. (m) Schematic diagram of signal acquisition for continuous wrist joint flexion at 30° and 60°.

We specifically applied FOCUS to monitor the deformation of finger joints and the periocular region, yielding excellent strain feedback. We first applied the FOCUS‐3D directly to a Vertical saccade task. First, we secure the FOCUS sensor to the upper eyelid using medical pressure‐sensitive adhesive. This adhesive offers excellent biocompatibility, making it the optimal choice for sensor fixation. After wearing FOCUS, the tester performs the relevant eye‐tracking tasks. The figure clearly demonstrates the testing process with FOCUS secured at the temple and eyelid position (Figure S17). We applied FOCUS‐3D directly to the vertical gaze task. By utilizing participants' 3D facial scans, the vertical gaze task was divided into four steps forming a cycle: Gaze Upward (Figure 6a); Gaze Straight Ahead (Figure 6b); Gaze Downward (Figure 6c); Eye Closure (Figure 6d).

The resulting physiological signals exhibited a high degree of regularity. Although the amplitude of eye movement is inherently difficult to control, the fluctuations present in the signal highlight the sensor's exceptional accuracy in capturing nuanced movements (Figure 6e). Crucially, subjects began exhibiting ocular fatigue during a sustained 5–10 s repeated vertical fixation task. The FOCUS sensor successfully captured this subtle change, revealing a slight signal attenuation occurring between 4 and 7 s into the task. Although the physiological range of motion of the human eye is uncontrollable, the overall task waveforms exhibited significant similarity, further validating FOCUS's high‐precision feasibility in complex eye‐tracking tasks. This technology holds promise for effective application in ADHD assessment and treatment, as FOCUS directly addresses the current limitations of ADHD evaluation methods—which are highly subjective, lack continuity, and lack physiological indicators. The subtle changes in the signal also offer a promising research direction for graded monitoring of eye fatigue.

Furthermore, experiments are divided into two parts. The first part aims to demonstrate its effective application in potential tasks such as eye tracking (Figure 6f–i), thereby providing more effective methods for eye tracking tasks and ADHD (Attention‐Deficit/Hyperactivity Disorder) monitoring and assessment [43, 44]. To be specific, FOCUS was deployed at the subject's upper eyelid and temple locations for signal capture (Figure S16). When subjects performed blinking and eye‐opening actions, FOCUS captured physiological signals reflecting different deformations. Despite the extremely minute deformations at the eyelid position (Figure 6f,g) and temple position (Figure 6h,i) caused by blinking, FOCUS was still able to capture physiological signals in real time.

Crucially, current eye tracking and ADHD assessment systems lack flexible sensors as monitoring devices, a significant weakness. Traditional eye tracking and ADHD assessment primarily rely on optical eye tracking systems. This method, based on infrared cameras and image processing, achieves high accuracy but has significant limitations. The equipment is bulky and requires a laboratory environment; its limited postural control makes it difficult to adapt to natural settings; its high cost and sensitivity to lighting conditions restrict both clinical and home applications. In contrast, FOCUS, a flexible wearable device that attaches to the periocular motor unit, enables continuous, non‐invasive, real‐time monitoring, providing a lightweight and flexible solution for eye tracking and ADHD assessment. As a potential flexible device, FOCUS can serve as a tool for monitoring eye movement functional units around the eye (Figure S16). We deployed FOCUS on the eyelids and temples to perform normal blinks and normal eye openings in computer tasks (Figure 6f,g). Clearly, the physiological signals collected from the temples differ from those collected from the eyelids (Figure 6h,i). Furthermore, the eyelid deformation caused by blinking is greater than that of the temple, demonstrating FOCUS's precise capture of periorbital ultra‐micromotion. The second part involves routine application to the finger joints and wrist as precision tests to assess FOCUS's sensitivity and accuracy. First, we attached the sensor to a finger joint (index finger) and used a professional rehabilitation joint angle measurement ruler to control the finger's flexion angle. We monitored the index finger's physiological signals at 0°, 15°, 30°, 45°, 60°, and 90°. The signal gradients resulting from these five angle changes were very stable (Figure 6j). Next, we tested the physiological signals of the index finger during continuous flexion at approximately 30° and 60°. The sensor potential change at 60° was significantly higher than that at 30° (Figure 6k). For the wrist, the physiological signals for free upward and downward movement (Figure 6l) and for continuous flexion at approximately 30° and 60° were highly significant and accurate (Figure 6m). Therefore, the finger joints and wrist, as joints with the smallest deformation and the highest number of movements among human joints, demonstrate the potential for FOCUS to accurately monitor movements as a skin sensor.

## 3 Conclusion

In this work, we introduce FOCUS, a novel paradigm for amplifying the sensitivity of LM sensors and enabling micro‐scale deformation monitoring. FOCUS inherently reimagines the fusion of structure and function, achieving significant breakthroughs in sensitivity, structural design, manufacturing, and application performance. Our design leverages the signal amplification properties of the Wheatstone bridge, combined with a spatially folded configuration, to transform a 2D planar structure into a 3D orthogonal. This unique innovation not only activates all four bridge arms, enabling positive sensitivity superposition—establishing a groundbreaking sensitivity amplification paradigm in the field of flexible sensors, but also dramatically enhances spatial efficiency, endowing the device with full‐axis strain capability and ultra‐high sensitivity. The 3D orthogonal structure represents a revolutionary, universally applicable spatial layout architecture for sensors. It ingeniously transforms traditional 2D planes into 3D frameworks, substantially lowering manufacturing barriers and facilitating mass production. In addition, as a universal sensor architecture, it is applicable to flexible materials such as LM and carbon nanomaterials. By arranging sensing elements in a 3D orthogonal layout, not only is sensitivity improved, but also temperature compensation is achieved through a differential mechanism, ensuring stability and high precision under complex conditions. In manufacturing, FOCUS draws inspiration from origami art, bypassing the complex multi‐layer stacking processes of traditional 3D flexible sensors. It enables mass production through simple screen printing and hot pressing, eliminating the need for expensive lithography and precision microfluidic technologies.

FOCUS achieves breakthroughs in key performance metrics: sensitivity at 50% strain exceeds LM sensors by over fivefold, with ultra‐fine response accuracy of 25 µm enabling sub‐millimeter and micrometer‐level detection tasks. FOCUS exhibits high stability (>5000 cycles) and excellent directionality (full‐axis strain detection), operating reliably across a broad temperature range from room temperature to 75°C. Through systematic comparison of key metrics—including material, sensitivity, directionality, dimensions, minimum resolution, manufacturing process, and stretchability—across various strain sensors in different studies (Table S2), FOCUS demonstrates significant combined advantages. Compared to other strain sensors fabricated from EGaIn materials, FOCUS achieves a GF value of 2.0—significantly higher than the typical GF range (0.49–1.81) and withstands extreme stretching up to 1000%. It simultaneously enables full‐axis, four‐directional strain monitoring, overcoming the limitation of existing EGaIn sensors that are generally confined to unidirectional monitoring. Unlike strain sensors made from other materials, FOCUS avoids performance trade‐offs: it maintains high sensitivity while delivering exceptional stretchability and directionality, overcoming the disadvantages of CNT‐based high GF materials in terms of stretchability (typically below 100%), directionality, and resolution. In contrast, FOCUS successfully avoided performance trade‐offs. Equally commendable is its fabrication process, which integrates simplicity, low cost, and high yield into a comprehensive advantage. Utilizing screen printing and innovative folding techniques, the process is significantly streamlined, bypassing costly or cumbersome methods like lithography or complex stacking. This facilitates large‐scale production and opens potential applications across multiple domains.

Employing LM as the conductive material, its universal 3D orthogonal structure overcomes the inherent low sensitivity of LM, achieving substantial performance enhancements that far surpass sensors relying solely on material properties or 2D structures. Essentially, FOCUS establishes a paradigm for universal sensor architectures, seamlessly integrating high sensitivity, omnidirectional detection, and simplified manufacturing. More persuasively, we successfully applied FOCUS to human joints and periocular regions, demonstrating exceptional tracking accuracy and response sensitivity in multi‐task eye‐tracking tests. This validates its significant potential for clinical diagnostic assistance and personalized interventions. This research not only provides a groundbreaking paradigm for LM sensors but also effectively overcomes their critical bottlenecks: inherent low sensitivity and the limitation of being unable to monitor biomechanical deformation at the microscopic scale.

## 4 Experimental Section

### 4.1 Fabrication of the MPC Ink

The fabrication of the liquid metal (LM) sensor begins with the preparation of metal‐polymer conductor (MPC) LM ink. The preparation of FOCUS is based on the preparation of MPC ink. Therefore, to obtain MPC ink, we added 1 g of PVP [polyvinylpyrrolidone; Mn (number‐average molecular weight) = 360 000, Aladdin, China] to 19 grams of hexanol (98%, Macklin, China) and stirred for 48 h to obtain a PVP solution. We used a probe ultrasonicator (S450D, Changsha Kun Yong Materials Co., Ltd., China) to ultrasonically treat 3 grams of gallium‐indium eutectic alloy (EGaIn, 75.5% gallium and 24.5% indium by weight, Changsha Kun Yong Materials Co., Ltd., China) in 1 mL of PVP solution at 20% amplitude for 60 s. (Branson, USA).

### 4.2 Fabrication of the Liquid Metal (LM) Sensor

First, we fabricated the designed LM sensor into a screen printing plate for screen printing. Then, we use the screen printing plate (325 mesh template) to print MPC ink on TPU film (with release liner, 3412, 1 mil, Bemis, USA) to obtain the LM sensor. After MPC printing, we dry the LM sensor prepared by MPC in an oven at 80°C (DHG‐9420A, Yiheng Scientific Instruments Co., Ltd., China) for 5 min. The LM sensor is non‐conductive after printing because the LM particles in the MPC ink are covered with an insulating oxide layer. Finally, we stretched the TPU substrate to 50% strain to activate the LM in the LM sensor. Since the strain caused by stretching the MPC ink breaks the insulating oxide layer and forms conductive pathways between the LM particles, the LM sensor becomes conductive and stretchable. Finally, a layer of TPU film is thermally bonded (150°C) over the dried LM sensor to encapsulate it, forming the LM sensor.

### 4.3 Fabrication of the FOCUS

We selected elastic thermoplastic polyurethane (TPU) as the substrate and encapsulation layer material. We subjected the Ga‐In alloy to ultrasonic treatment in a polyvinylpyrrolidone (PVP) solution to obtain LM ink (MPC, ref. [42]), and screen‐printed this ink onto the TPU substrate. After encapsulation with TPU film, we obtained FOCUS‐2D (Figure 2a). To activate the LM particles, we applied strain (approximately 20%) along two axes of the LM‐printed substrate to break the oxide layer of the LM particles and form conductive pathways between them.

We divide the Fabrication steps for FOCUS into two parts: FOCUS‐2D and FOCUS‐3D. The MPC ink is screen‐printed onto the TPU film (with release film, 3412, 1 mil, Bemis, USA) according to the preparation process to obtain the FOCUS (Figure 2a). Then, we dry it in an oven (DHG‐9420A, Yiheng Scientific Instruments Co., Ltd., China) at 80°C for 5 minutes. Finally, a layer of TPU was thermally pressed to complete the FOCUS preparation. Finally, we used a laser cutter (355 nm Nd: YAG laser, 5 W, Shenzhen JPT Optoelectronics Co., Ltd., China) to cut the outer contour of FOCUS‐2D at a marking speed of 60 mm s^−1^ to obtain a windmill‐shaped sensor. The FOCUS‐3D preparation process follows the folded procedure, with two folds (Figure 1a). Then, we completed the sensor preparation through once hot pressing (up to 150°C) process. Finally, based on the Wheatstone bridge principle and sensor pin design, we connected an Arduino microcontroller and signal output externally.

### 4.4 Fabrication of the LM sensor and Half Bridge Sensor

The LM Sensor is prepared by directly connecting the LM sensor to the Wheatstone bridge, with the other three remaining as resistors. The LM sensor is connected to the R1 position, R2 is 30 ohm, and R3 and R4 are 3k ohm. For the Half Bridge Sensor, we designed a double‐bridge structure using CAD, then followed the aforementioned hot pressing to hot‐press the two prepared LM sensors in perpendicular directions and connect them to the Wheatstone bridge to replace the R1 and R2 resistors. The R3 and R4 resistors are 3k ohm, respectively.

### 4.5 Characterization of the FOCUS

We used a programmable linear guide block (FSL_40, FUYU, China) to stretch the sample at a speed of 100 mm/min for tensile testing and at a speed of 1000 mm/min for cyclic testing to evaluate the sensor performance. Second, the two ends of the test sample were fixed to a universal testing machine (XLD‐1000E, JINGKONG, Guangzhou, China) using fixtures. The universal testing machine was used to apply uniform tensile stress to the sample via computer software, simultaneously acquiring stress and strain data to generate corresponding stress‐strain curves.

The sensor's resistance was measured using a multimeter (Fluke F106, China). The sensor was laser‐cut using a laser cutter (YaJu Laser, 5W, China). We used an electrochemical station (1040C, CHI, China) to record the resistance in real‐time during tensile testing and cyclic testing. The surface morphology of FOCUS was characterized using a scanning electron microscope (SEM Scan, CIQTEK, SEM5000Pro). In the application experiment, sensor signals were collected via an integrated circuit board and transmitted to a computer via Bluetooth for data storage and analysis. Additionally, the FOCUS sensor underwent surface testing on participants' skin (all participants signed informed consent forms), and this test was approved by the institutional ethics committee (Medical Ethics Committee of Capital Medical University, 2021SY021).

## Author Contributions

Z.W. and L.T. conceived and supervised this project. Z.W. designed, fabricated, and characterized the FOCUS. Z.W. wrote the manuscript. All authors discussed the results and commented on the manuscript.

## Conflicts of Interest

The authors declare no conflict of interest.

## Supporting information




**Supporting File**: advs73611‐sup‐0001‐SuppMat.docx.

## Data Availability

The data that support the findings of this study are available from the corresponding author upon reasonable request.
