# Supplementary material for: FOCUS: A Four‐In‐One Consolidated Unison Strain Sensor with Enhanced Sensitivity
Source: Adv Sci (Weinh). 2026 Jan 27;13(14):e19613. doi: 10.1002/advs.202519613 (PMC12970242; doi:10.1002/advs.202519613)
Supplement: Supplementary file 1 — Supporting File: advs73611‐sup‐0001‐SuppMat.docx. [file ADVS-13-e19613-s001.docx]

**Supporting Information**

Title: FOCUS: A Four-in-One Consolidated Unison Strain Sensor with Enhanced Sensitivity

Zimeng Wang^1^, Ruiran Li^1^, Bowen Yang^1^, Zhuofei Peng^1^, Muyang Jing^1^, Yu Gu^1*^, and Lixue Tang^1,2*^

The file includes:

Figure S1 to S18

Figure S1: Schematic diagram of the position and structure of the FOCUS 2D planar linear displacement sensor.

Figure S2: Spatial layout diagram of the foldable FOCUS and top view of the folding process.

Figure S3: FOCUS scanning electron microscopy characterization.

Figure S4: Schematic diagram of the LM sensor stretching principle.

Figure S5: LM Sensor Sensitivity Fitting Curve Diagram.

Figure S6: FOCUS Sensitivity Amplification Mechanism Diagram.

Figure S7: Schematic diagram of the physical stretching of the LM sensor.

Figure S8: Schematic diagram of four different sizes of LM sensors.

Figure S9: Mechanical test diagram.

Figure S10: Diagram of a LM sensor electrical connection.

Figure S11: Diagram of a half-bridge electrical connection.

Figure S12: Side-by-side comparison of sensor sensitivity across three configurations.

Figure S13: FOCUS gradient tensile––recovery experiment.

Figure S14: FOCUS Response Time Chart.

Figure S15: FOCUS Temperature Cycling-Strain Dynamic Experiment.

Figure S16: Diagram of FOCUS deployment in the periorbital area.

Figure S17: The Eyelid structure and FOCUS layout.

Figure S18: Schematic diagram of the FOCUS Wheatstone bridge circuit design.

Table S1: LM Sensor and FOCUS Performance Comparison.

Table S2: Comparison of sensitivity improvement strategies for different flexible strain sensors.

**Other Supporting Material for this manuscript includes the following:**

Supplementary Note 1: Principle of LM sensor tensile.

Supplementary Note 2: The signal amplification principle of the Wheatstone bridge.

**
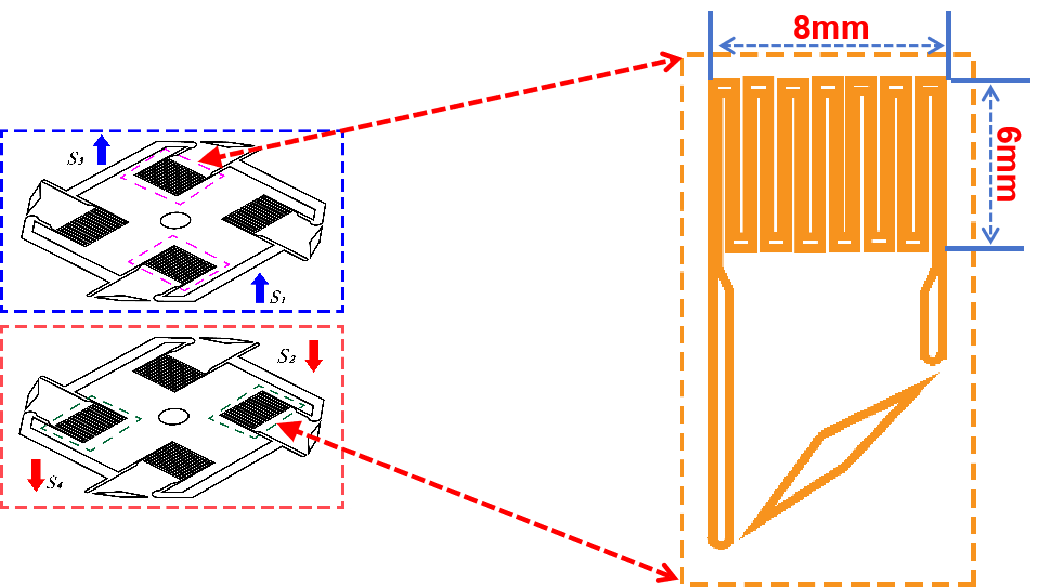
**

**Figure S1: Schematic diagram of the position and structure of the FOCUS 2D planar linear displacement sensor.** The left image shows the distribution of four LM sensors within the FOCUS system. The right image displays the LM sensor dimensions: 8 mm in length and 6 mm in width.


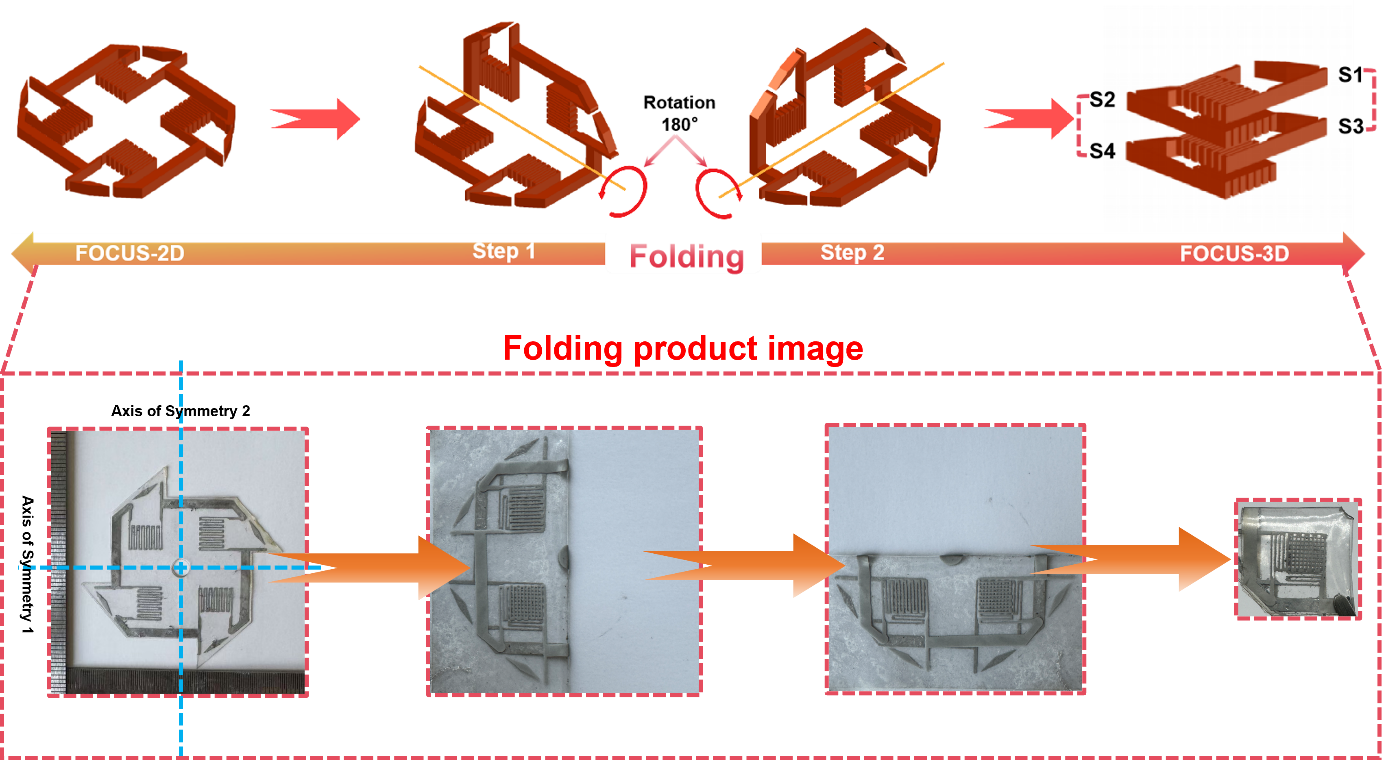


**Figure S2: Spatial layout diagram of the foldable FOCUS and top view of the folding process.** FOCUS-3D forms spatial configurations S1, S2, S3, and S4. The figure also shows the sensor's top-down perspective during the transformation from a 2D plane to a 3D device.


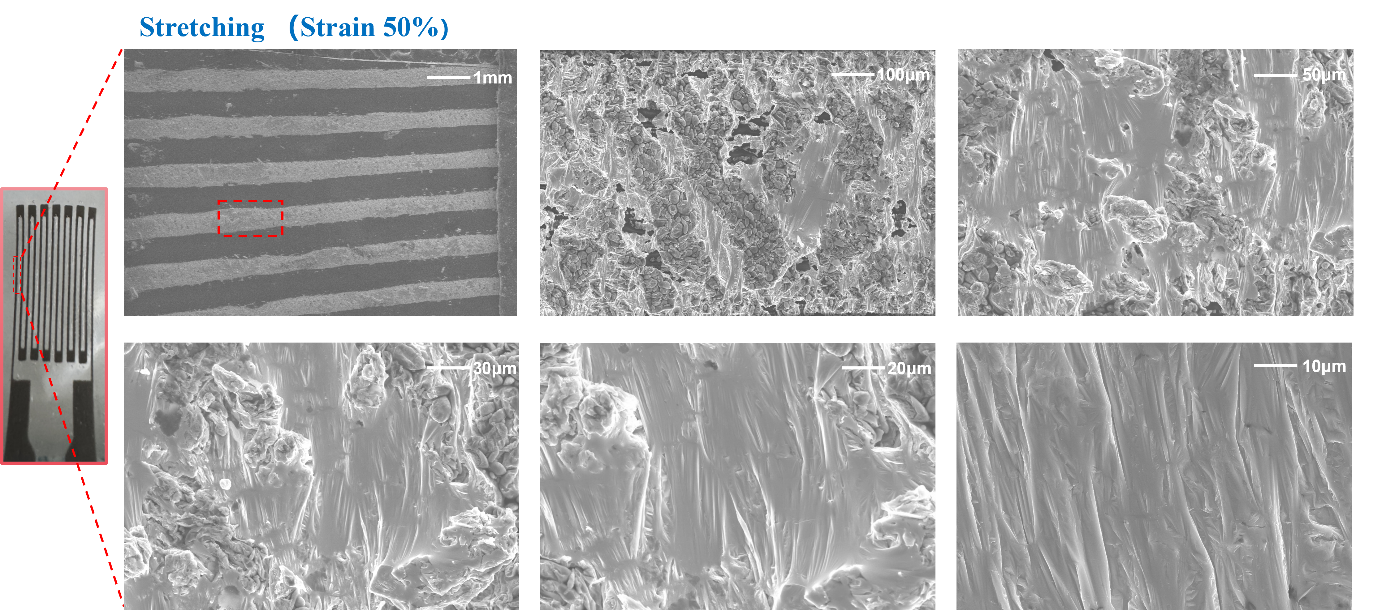


**Figure S3: FOCUS scanning electron microscopy characterization.** SEM images of FOCUS from pre-tensile to tensile to post-tensile states. After tensile deformation, liquid metal particles in FOCUS achieve complete sintering, exhibiting excellent electrical conductivity.


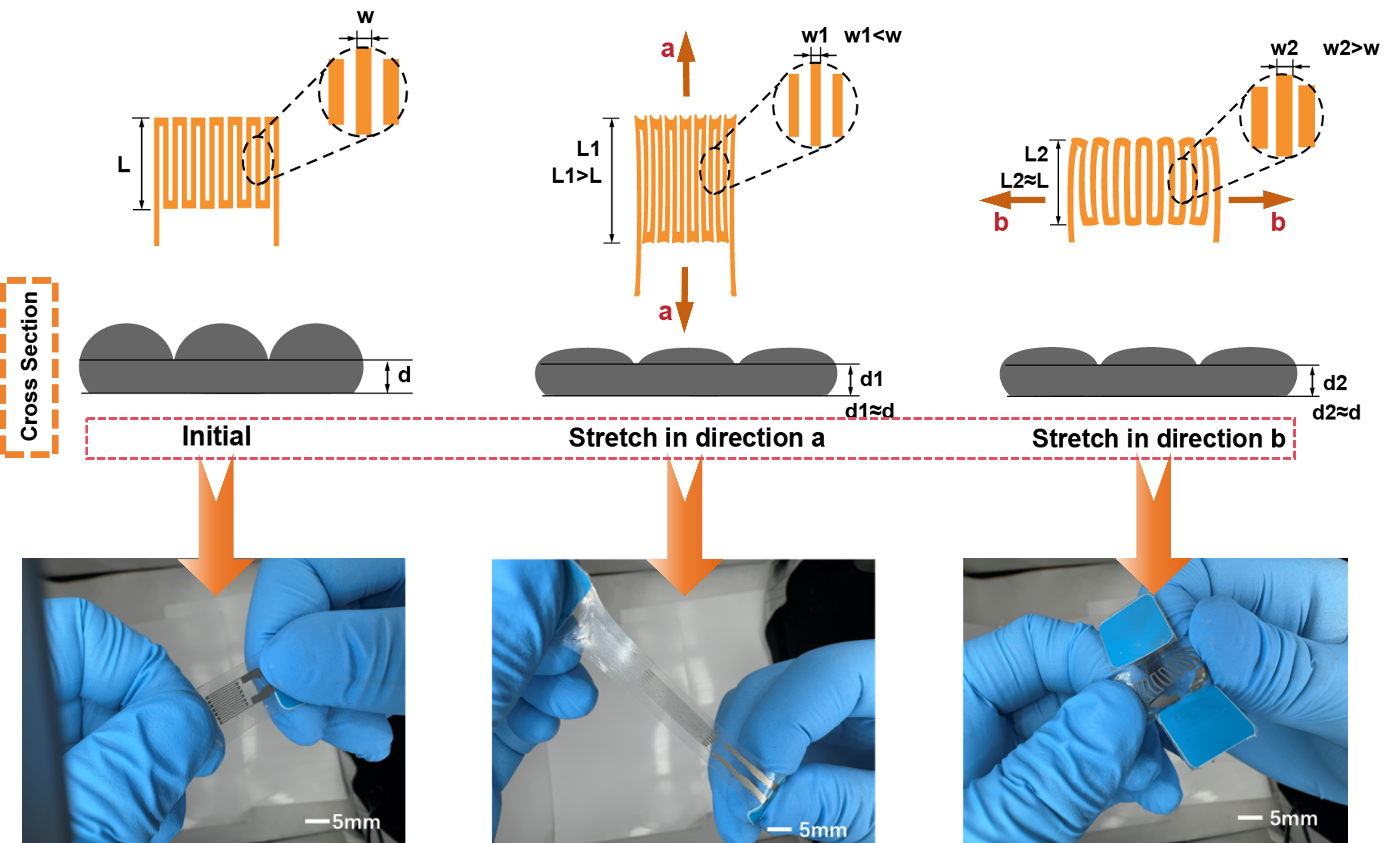


**Figure S4: Schematic diagram of the LM sensor stretching principle.** We define the longitudinal and transverse stretching directions of the sensor as **a and b,** respectively.


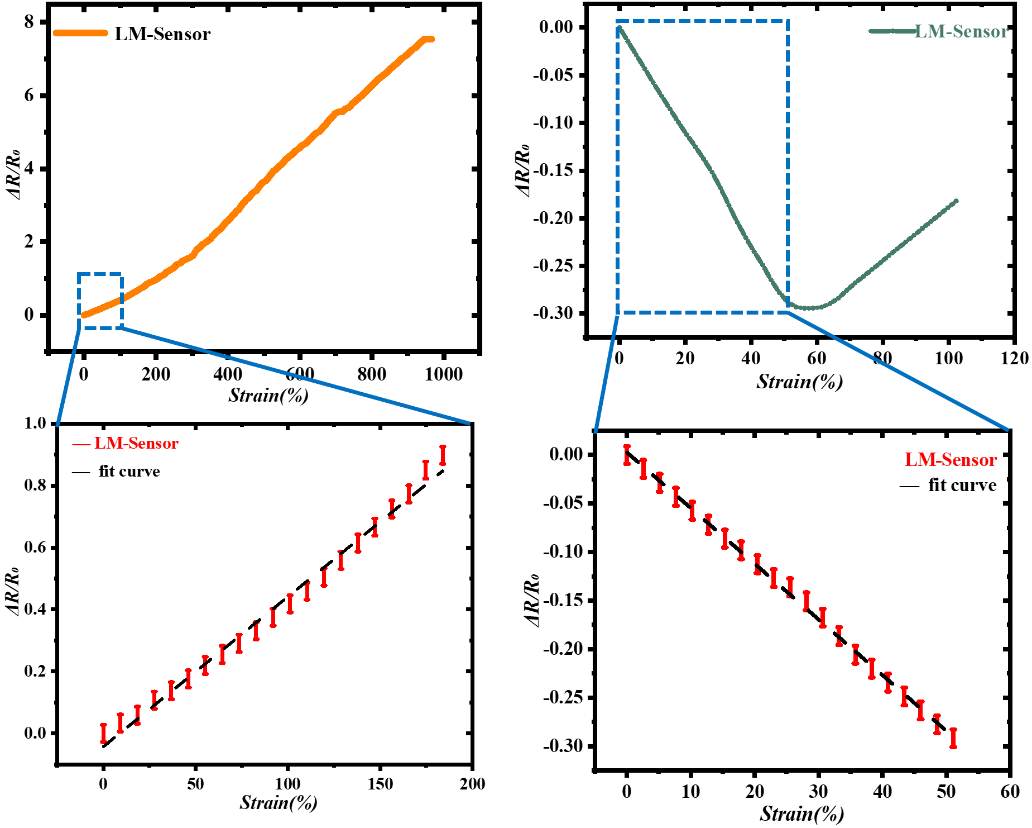


**Figure S5: LM Sensor Sensitivity Fitting Curve Diagram.** The LM sensor exhibits excellent linearity during tensile testing in both the a and direction bs, with R² values of 0.991 and 0.998 respectively.


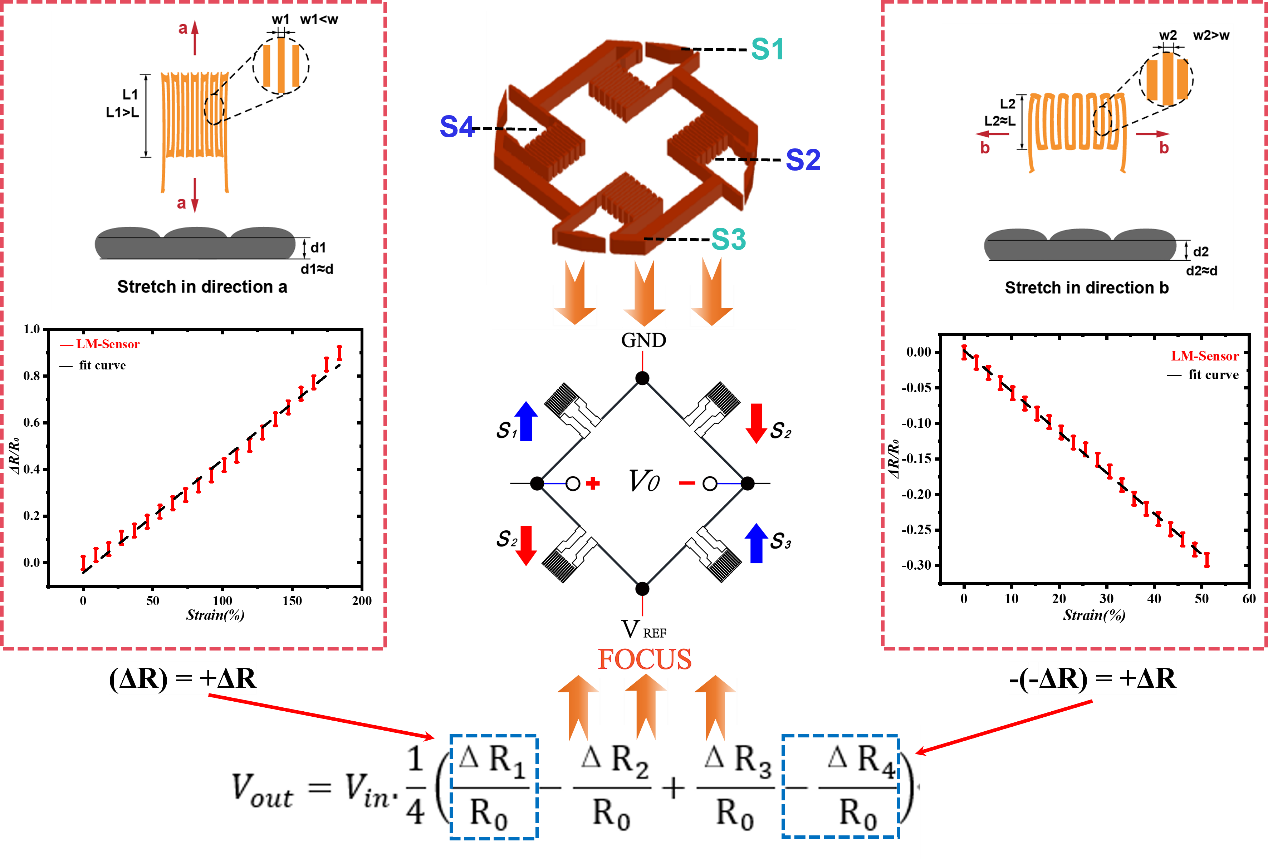


**Figure S6: FOCUS Sensitivity Amplification Mechanism Diagram.** The sensitivity amplification mechanism of FOCUS primarily combines the tensile principle of LM sensors with the differential principle of the Wheatstone bridge.


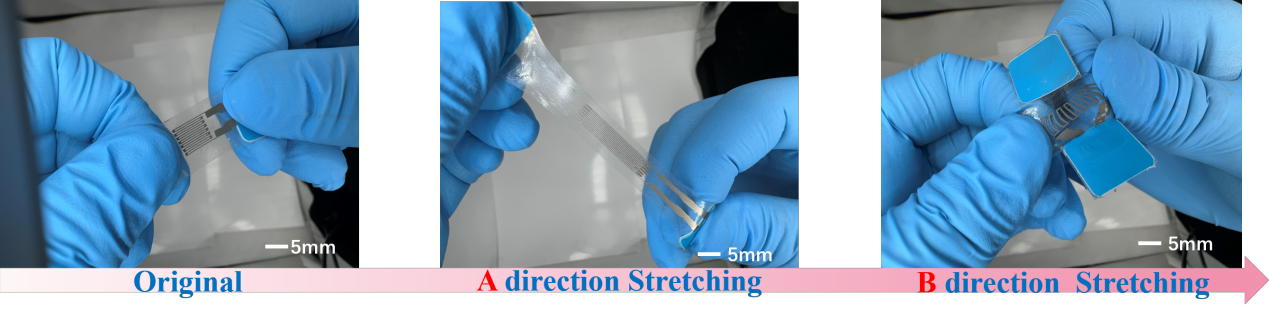


**Figure S7: Schematic diagram of the physical stretching of the LM sensor.**


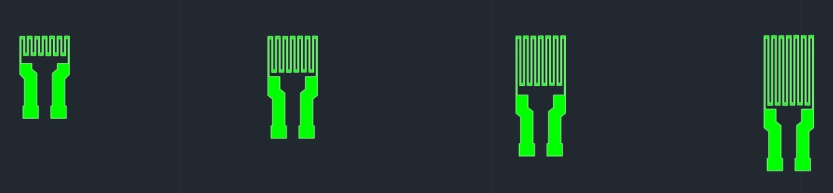


**Figure S8: Schematic diagram of four different sizes of LM sensors.** Four sizes of LM sensors were used to experiment on the effect of size on sensitivity. The length of the vertical axis increases from left to right, and the second one is the LM sensor.


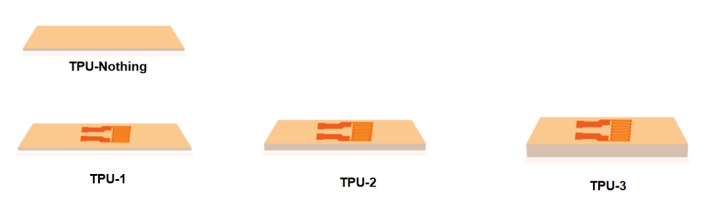


**Figure S9: Mechanical test diagram.** The blank TPU(TPU-Nothing) was used for comparison with TPU-1 (printed LM sensor) of the same thickness; TPU-1, TPU-2, and TPU-3 were used for comparison with TPU of different thicknesses. TPU1, 2, 3 thickness gradually increases.


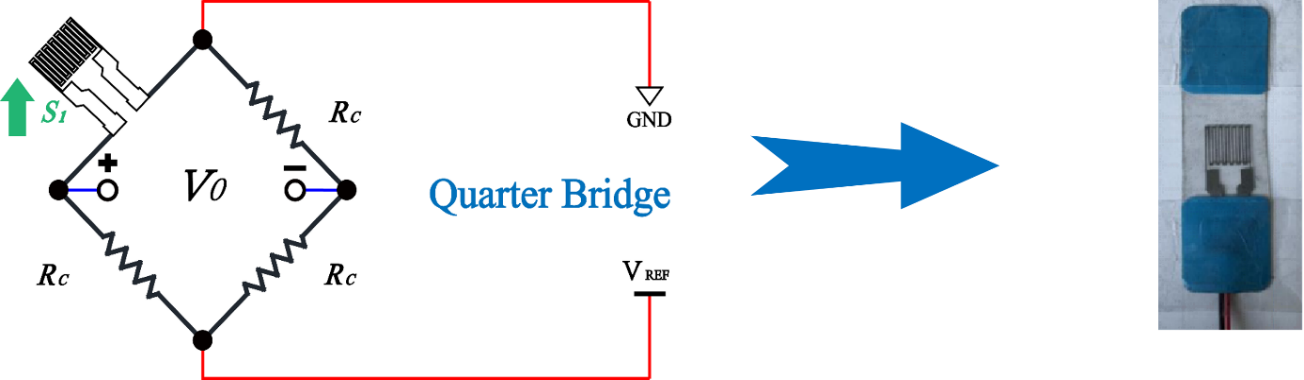


**Figure S10: Diagram of a LM sensor electrical connection.** The quarter-bridge sensor is connected to the Wheatstone bridge as S1.


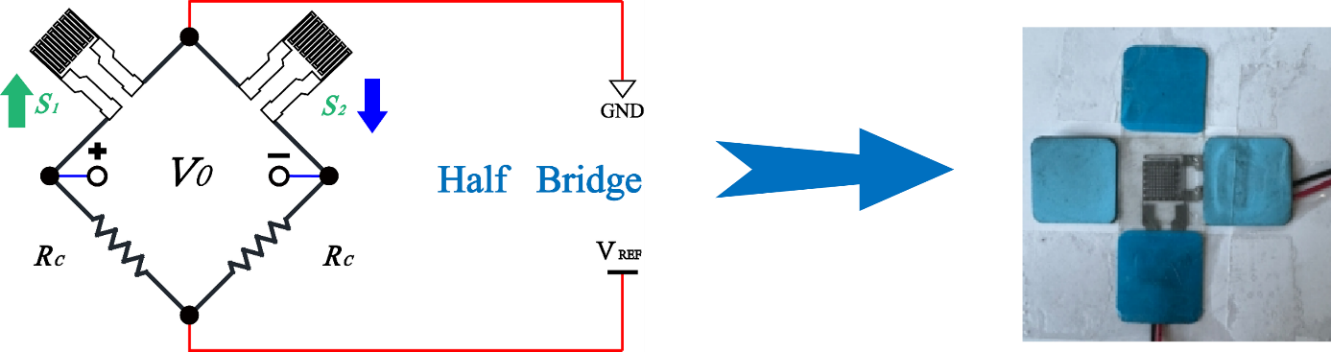


**Figure S11: Diagram of a half-bridge electrical connection.** The half-bridge sensor is connected to the Wheatstone bridge as S1 and S2, and S1 and S2 are arranged in an orthogonal manner.

**
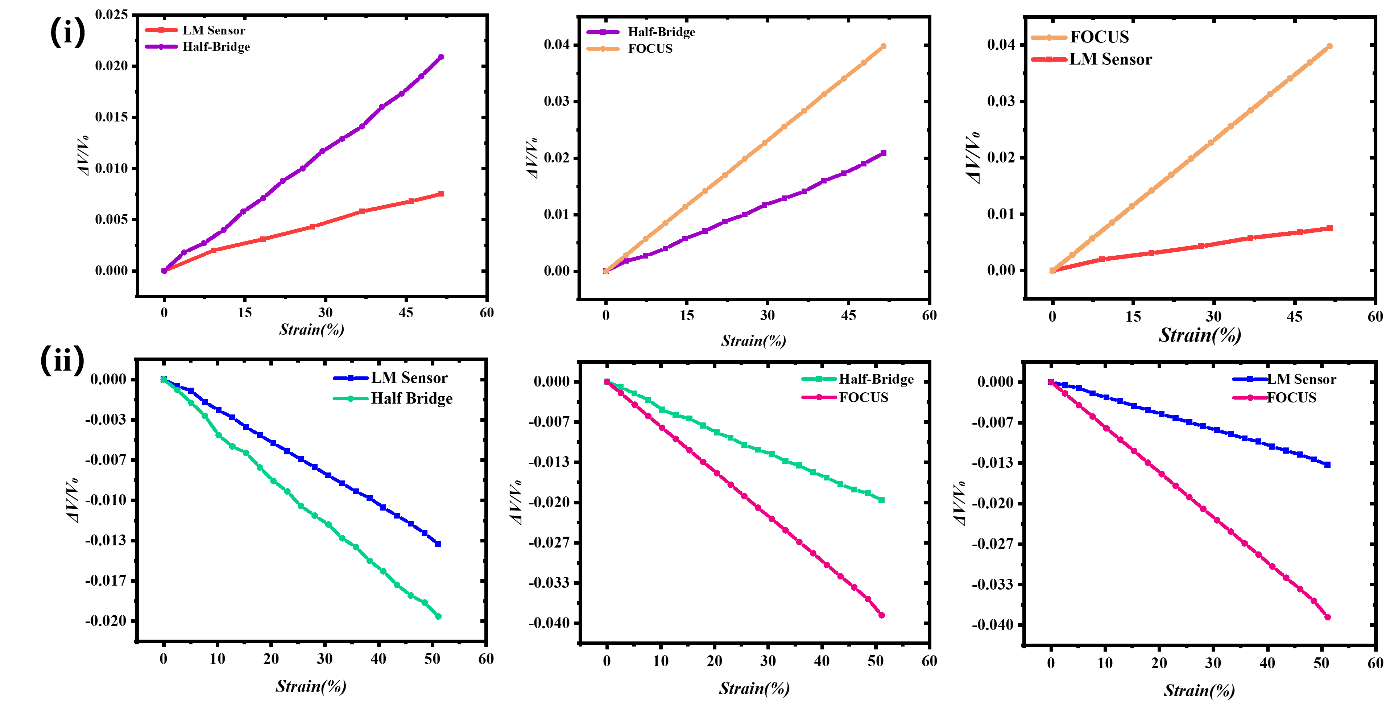
**

**Figure S12: Side-by-Side Comparison of Sensor Sensitivity Across Three Configurations.** Clearly and completely reflects the quantitative relationships between each pair of the three bridge arm sensors: **i** represent tensile forces in direction a; **ii** represent tensile forces in direction b.

**
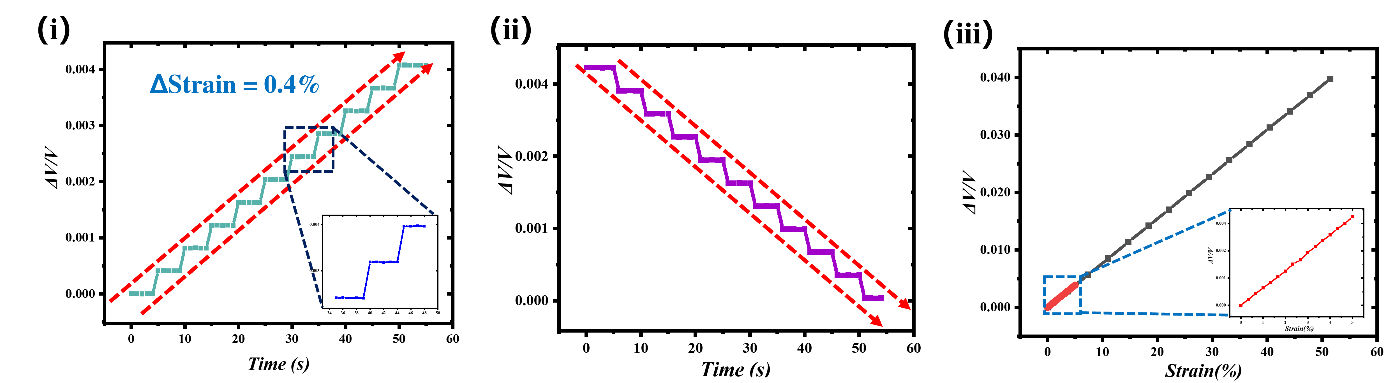
**

**Figure S13: FOCUS gradient tensile––recovery experiment. (**i) is the FOCUS temperature cycling diagram; (ii) shows dynamic cycling strain and contraction sensitivity images of FOCUS at different temperatures; (iii) depicts FOCUS completing 50% strain cycling experiments.


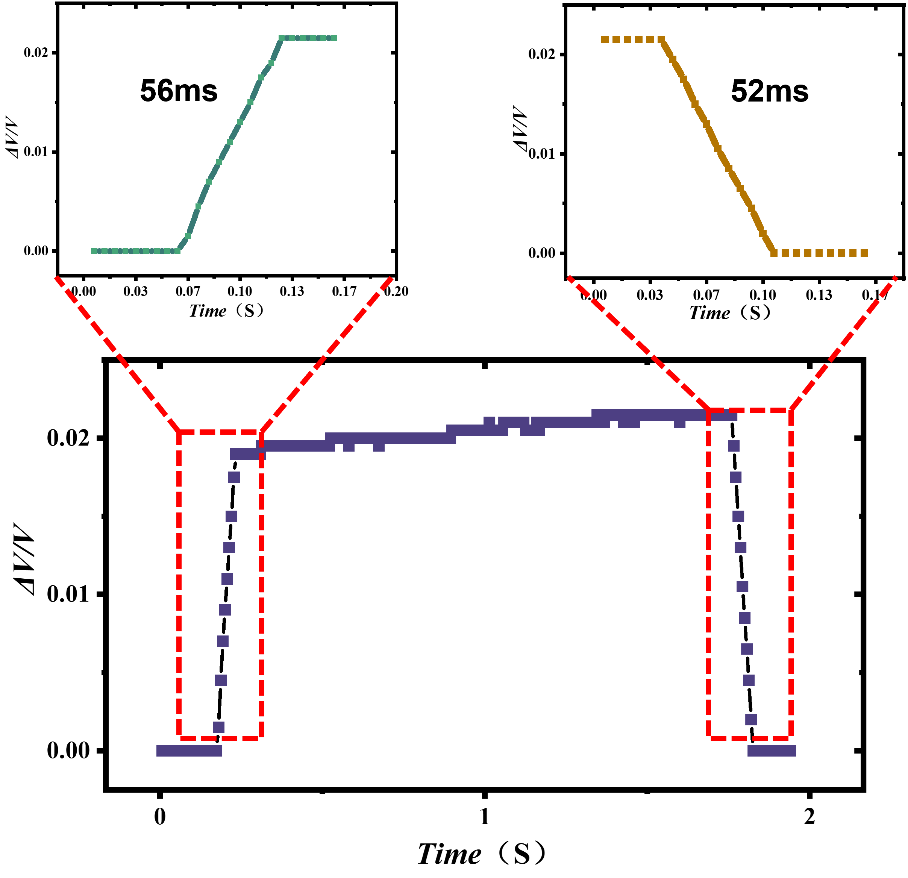


**Figure S14: FOCUS Response Time Chart.** FOCUS response time and recovery time at 5 % strain and 200 mm/s tensile speed rate.


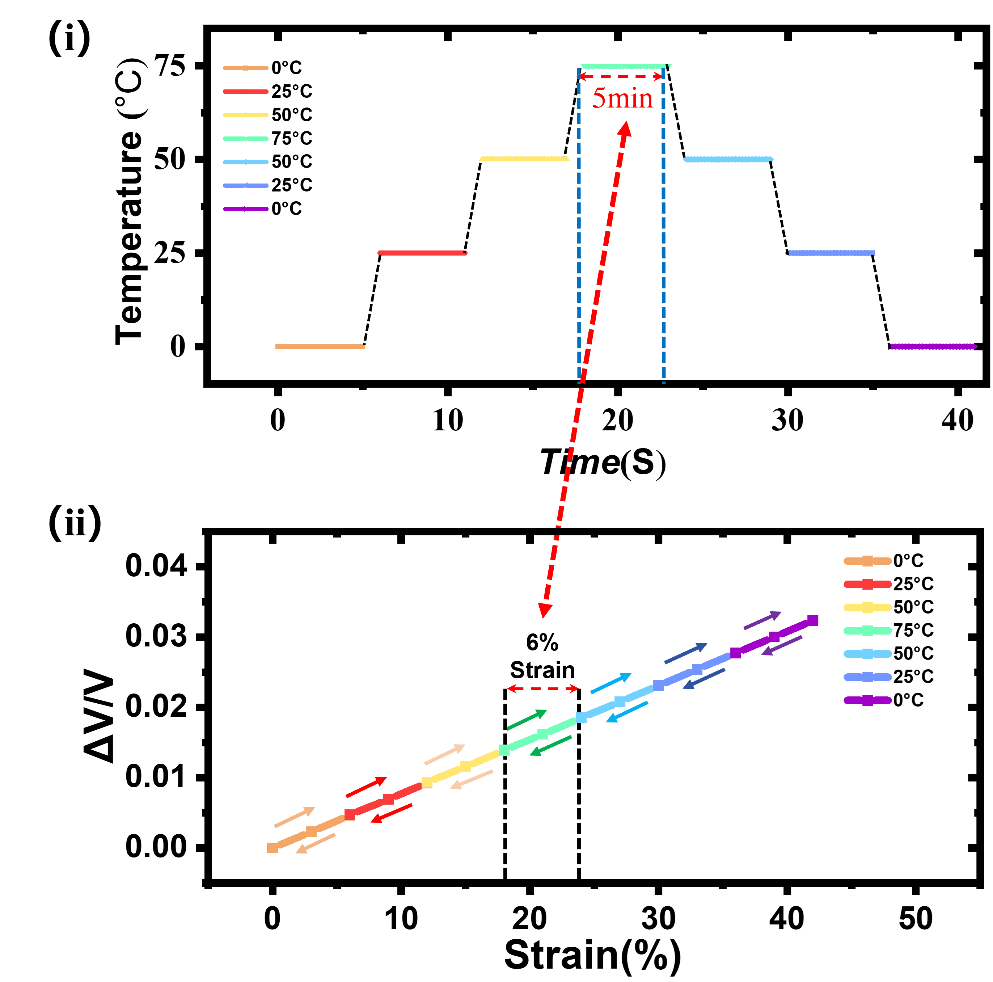


**Figure S15: FOCUS Temperature Cycling-Strain Dynamic Experiment.** (i) is the FOCUS temperature cycling diagram; (ii) shows dynamic cycling strain and contraction sensitivity images of FOCUS at different temperatures.


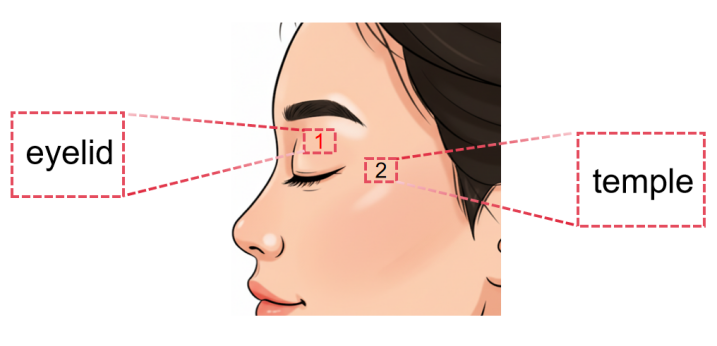


**Figure S16: Diagram of FOCUS deployment in the periorbital area.** We deployed FOCUS at the eyelids (position 1) and temple (position 2) for monitoring.

**
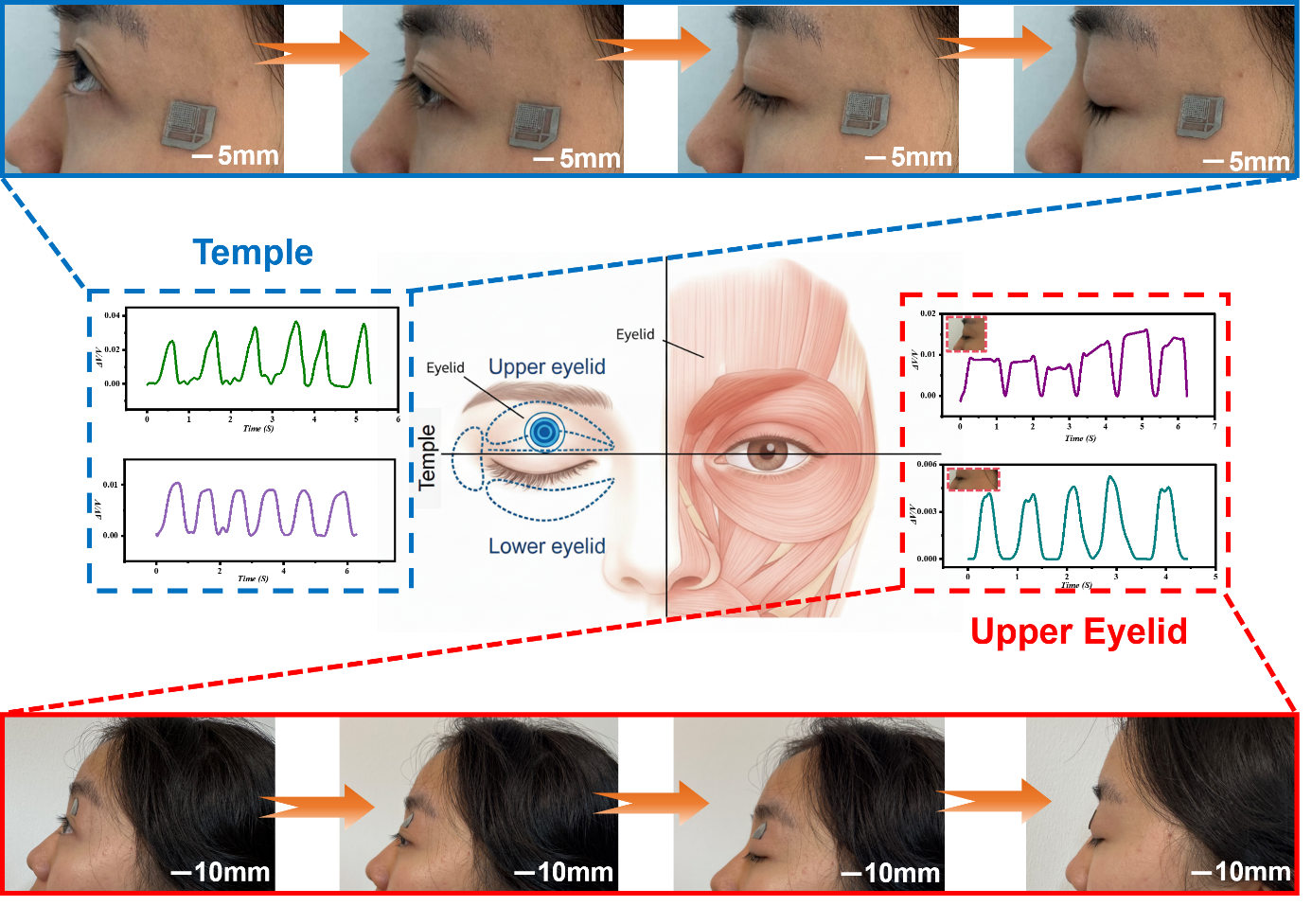
**

**Figure S17:** **The Eyelid structure and FOCUS layout.** While conventional eye-tracking primarily measures eyeball motion, a deeper physiological link exists between eye movement and the surrounding musculature.

Every eye movement is precisely governed by a network of muscles that are responsible for both eye and eyelid actions. This relationship is a core tenet of our work:

**Eyelid-Eye Movement Synergy:** Muscles such as the levator palpebrae superioris and the orbicularis oculi work in close synergy with the eye itself. These muscles control eyelid opening and closing, and their activity provides a direct physiological record of blinking and other subtle eye dynamics.

**The Temple Region as a Biomarker:** The muscle group located in the temple region does not directly control eye movement, but it is highly reactive to cognitive load and mental stress. Its subtle contractions during focused tasks or during periods of fatigue can serve as a powerful physiological biomarker.

By strategically deploying our FOCUS sensor on both the eyelid and the temple, we move beyond simple behavioral tracking. This strategic placement allows us to directly decode physiological signals from their muscular source. The result is high-fidelity data that can be used for a deeper analysis of cognitive states and pathologies.

**
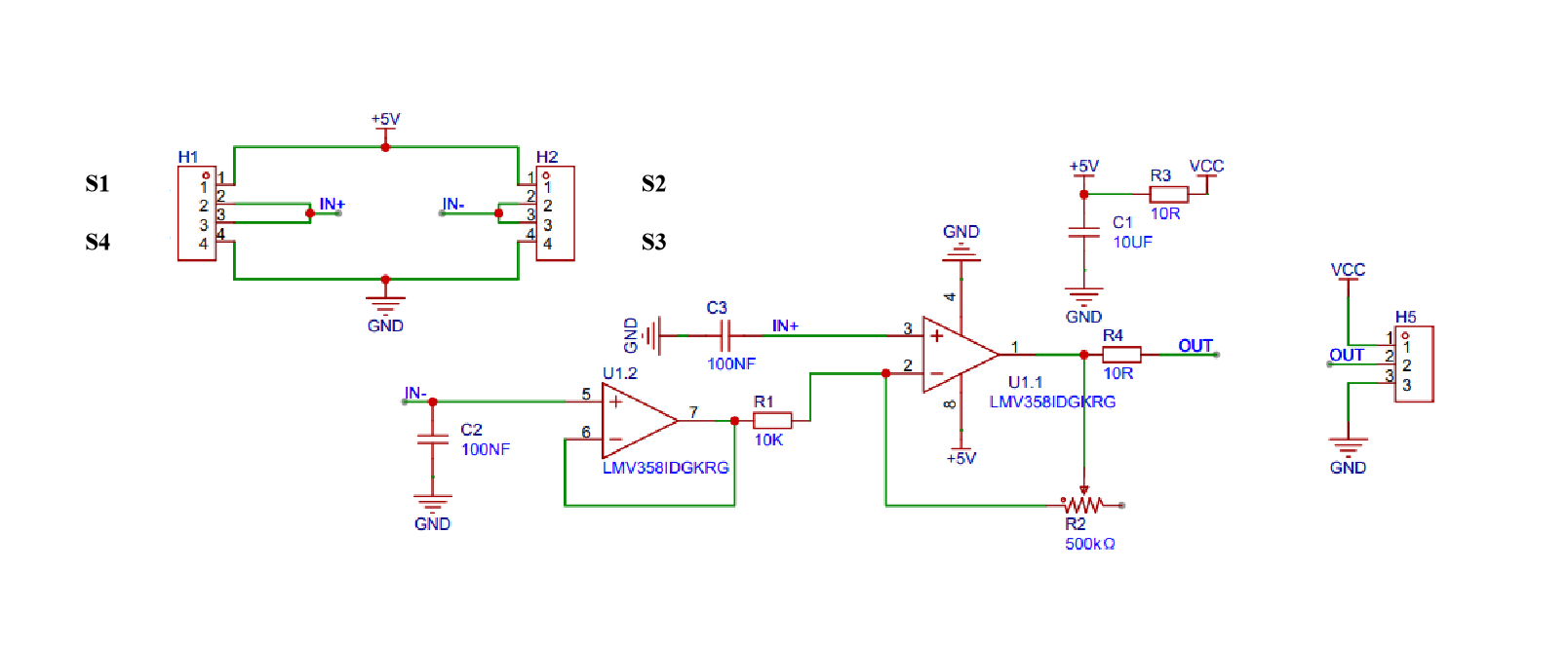
**

**Figure S18: Schematic diagram of the FOCUS Wheatstone bridge circuit design.** The figure illustrates the Wheatstone bridge integrating four LM sensors as the equivalent FOCUS for full-bridge experiments.

**Table S1: LM Sensor and FOCUS Performance Comparison.**

| Parameters | LM Sensor | FOCUS | Lifting Multiplier |
| --- | --- | --- | --- |
| Sensitivity | $\text{V}_{\text{out}}\text{=}\text{V}_{\text{in}}\text{⋅}\frac{\text{Δ}\text{R}}{4\text{R}_{\text{0}}}$ | $\text{V}_{\text{out}}\text{=}\text{V}_{\text{in}}\text{⋅}\frac{\text{Δ}\text{R}}{\text{R}_{\text{0}}}$ | Significantly improved |
| Temperature Compensation | Incomplete, requires additional compensation circuitry | Fully Self-Compensating | More than  fivefold |

**Table S2: Comparison of sensitivity improvement strategies for different flexible strain sensors.**

| Ref. | Key  materials |  | Gauge  factor | Strain  Directionality | Size | Fabrication | Resolution | Stretchability |
| --- | --- | --- | --- | --- | --- | --- | --- | --- |
| This work | **EGaIn** |  | **GF=2** | **Full axial**  **strain, Four directions** | **8mm*8mm** | **Screen printing + folding** | **25 µm** | **~1000%** |
| 1 | Si-NRs |  | GF=0.4 | Omnidirectional strain, 8 directions | 1.3cm*1cm | Transfer printing + Lithography technology | N/A | ~10% |
| 2 | MWCNTs |  | GF=4.18 | Triple direction (0◦,45◦, 90◦) | 20 mm * 20 mm | Direct Ink Writing | N/A | N/A |
| 3 | EGaIn |  | GF=0.49 | Single Direction | N/A | Spray patterning | N/A | N/A |
| 4 | EGaIn |  | GF=1.81 | Single Direction | N/A | Atomized Spraying Process | N/A | 460% |
| 5 | EGaIn |  | 0.5 | Single Direction | N/A | N/A | N/A | 650% |
| 6 | EGaIn |  | 0.7 | Single Direction | N/A | N/A | N/A | N/A |
| 7 | EGaIn |  | 1 | Single Direction | N/A | Magnet patterning preparation | N/A | 300% |
| 8 | EGaIn |  | 1.2 | Single Direction | N/A | N/A | N/A | 300% |
| 9 | Au film |  | GF=0.02 | N/A | 2mm*2mm | Planar precursor fabrication +3D structuring | ~1.8 mm | ~20% |
| 10 | Silicon |  | GF=  0.07~0.5 | N/A | N/A | N/A | ~62.8 mm | 15~30% |
| 11 | Conductive hydrogel |  | GF=  1.24~4.15 | Single Direction | N/A | self-sorting | N/A | ~487% |
| 12 | PLLA |  | GF=3.3 | N/A | N/A | N/A | ~1 cm | ~15% |
| 13 | AgNWs |  | GF=5 | N/A | N/A | N/A | ~15 mm | ~60% |
| 14 | CNT/CB |  | GF=8 | Triple Direction | N/A | N/A | ~18 mm | ~35% |
| 15 | MXene |  | GF=8767.4 | N/A | N/A | N/A | N/A | ~83% |

In Table S2, Si-NRs as Ultra-thin Monocrystalline Silicon Nanoribbons; MWCNTs as Multi-walled carbon nanotubes; PLLA stands for polylactic acid; AgNWs represents as Agnanowires; CNT/CB stands for Carbon nanotube with carbon black.

**Supplementary References**

1. B. Hu, D. Xu, Y. Shao, Z. Nie, P. Liu, J. Li, L. Zhou, P. Wang, N. Huang, J. Liu, Y. Lu, Z. Wu, B. Wang, Y. Mei, M. Han, R. Li, E. Song, Sci. Adv. 2024, 10, eadp8804.

2. L. Yang, C. Hu, W. Hu, Z. Wang, M. Zhang, Y. Cang, B. Yang, Chemical Engineering Journal 2024, 502, 158115.

3. J. W. Kim, S. Kim, J. Lee, Y. Kim, J. S. Ha, Adv Funct Materials 2025, 35, 2411786.

4. T. Dong, J. Wang, X. Lai, C. Zhang, H. Li, IEEE Sensors J. 2025, 25, 7323.

5. Y. Duan, Z. Sun, Q. Zhang, Y. Dong, Y. Lin, D. Ji, X. Qin, *Nat Commun* **2025**, *16*, 6362.

6. H. Kim, G. Kim, J. H. Kang, M. J. Oh, N. Qaiser, B. Hwang, *Adv Compos Hybrid Mater* **2025**, *8*, 14.

7. J. Zhang, X. Zou, Z. Li, C. P. Y. Chan, K. W. C. Lai, *ACS Appl. Mater. Interfaces* **2025**, *17*, 6957.

8. S. Hou, H. Zhao, Q. Xu, D. Liu, G. Jin, Y. Xu, J. Wang, M. Kuang, X. Zhang, *J. Mater. Chem. C* **2025**, *13*, 17882.

9. Z. Liu, X. Hu, R. Bo, Y. Yang, X. Cheng, W. Pang, Q. Liu, Y. Wang, S. Wang, S. Xu, Z. Shen, Y. Zhang, Science 2024, 384, 987.

10. L. Xu, S. R. Gutbrod, A. P. Bonifas, Y. Su, M. S. Sulkin, N. Lu, H.-J. Chung, K.-I. Jang, Z. Liu, M. Ying, C. Lu, R. C. Webb, J.-S. Kim, J. I. Laughner, H. Cheng, Y. Liu, A. Ameen, J.-W. Jeong, G.-T. Kim, Y. Huang, I. R. Efimov, J. A. Rogers, Nat Commun 2014, 5, 3329.

11. Z.-Q. Gao, C.-H. Liu, S.-L. Zhang, S.-H. Li, L.-W. Gao, R.-L. Chai, T.-Y. Zhou, X.-J. Ma, X. Li, S. Li, J. Zhao, Q. Zhao, 2024.

12. C. M. Boutry, Y. Kaizawa, B. C. Schroeder, A. Chortos, A. Legrand, Z. Wang, J. Chang, P. Fox, Z. Bao, Nat Electron 2018, 1, 314.

13. J. H. Lee, S. H. Kim, J. S. Heo, J. Y. Kwak, C. W. Park, I. Kim, M. Lee, H. H. Park, Y. H. Kim, S. J. Lee, S. K. Park, Adv. Mater. 2023, 35, 2208184.

14. L. Zhang, S. Xing, H. Yin, H. Weisbecker, H. T. Tran, Z. Guo, T. Han, Y. Wang, Y. Liu, Y. Wu, W. Xie, C. Huang, W. Luo, M. Demaesschalck, C. McKinney, S. Hankley, A. Huang, B. Brusseau, J. Messenger, Y. Zou, W. Bai, Nat. Commun. 2024, 15, 4777.

15. X. Shi, H. Wang, X. Xie, Q. Xue, J. Zhang, S. Kang, C. Wang, J. Liang, Y. Chen, ACS Nano 2019, 13, 649–659.

**Supplementary Note 1: Principle of LM sensor tensile.**

The FOCUS sensor is a four-in-one single-bridge sensor. Essentially, it's a resistive strain sensor based on liquid metal particles. Its operating principle is that strain causes a change in resistance. When the liquid metal in the single-bridge sensor deforms under mechanical stress (tension or compression), the corresponding resistance changes. This change can be measured to calculate and confirm the applied strain. The change in sensor resistance is proportional to the applied strain. This change is expressed according to Ohm's law: Equation 1 (where R is the resistance, L is the conductor length, and A is the cross-sectional area).

$$R=\rho L/A$$

The calculation formula for the sensitivity index GF of the liquid metal strain sensor is as follows, where is the strain of the sensor.

$$GF=\frac{\Delta R/R_{0}}{\varepsilon}$$

The relationship between strain and length change the core of the strain formula is to relate the change in length ($\text{ΔL}$) to the original length (L0).

$$\text{ϵ=}\frac{\text{ΔL}}{\text{L}_{\text{0}}}$$

Bridge: a quarter bridge, a half bridge, and FOCUS. These three types formed three sets of experiments to monitor the sensitivity curves of the sensors under the same strain and compare them with each other. We used an Arduino NUO as a micro controller and also configured a differential amplifier to detect small voltage changes and amplify the signal. Figure S8 shows a single-bridge sensor configuration, using a single-bridge sensor S1 (30 ohms) and three commercial fixed resistors (R3 and R4, 3k ohms; R2, 30 ohms). The single-bridge sensor was fixed horizontally on the guide rail and generated axial strain along the Y-axis at a speed of 2mm/s. The real-time output voltage V out returned by the system was then recorded and the voltage difference was calculated:

$$\Delta V=v_{out} -v_{0}$$

At this point, we have calculated the linear correlation between the output voltage change of the sensor and the applied strain according to Formula 2, which is expressed as the sensor sensitivity:

$$GF=\frac{v_{out} -v_{0}}{\varepsilon}$$

In Figure S4，Analysis of the Three States of LM sensor:

**Initial State**: At this point, the sensor is in a mechanically relaxed state. Electrical State: The sensor resistance R_0_ is at its initial value, $\Delta R = 0$. Therefore:

$$\frac{\Delta R}{R_{0}}=0$$

**Longitudinal Stretch Principle (Direction a)**: Positive GF Response Mechanism. Longitudinal stretch occurs along the primary stretch axis of the LM path, activating traditional strain coupling and geometric amplification modes. During longitudinal stretch (Direction a), macroscopic strain is effectively transmitted along the primary axis of the LM path (Direction a), and the sensor exhibits a traditional positive GF response.

Macroscopic deformation: The structural length increases from L to L1 (L1 > L). Macroscopic strain is efficiently transmitted along the conductive path.

Micro LM Path Changes: Length Change: The LM path is effectively elongated, with relative length increasing: $\frac{\Delta L}{L_{0}}>0$. Cross-sectional Area Change: Following Poisson's ratio, the cross-sectional area A of the LM path decreases: $\frac{\Delta A}{A_{0}}<0$. Based on the above,

$$\frac{\Delta R}{R_{0}}> 0$$

LM exhibits positive GF response.

**Lateral Stretch Principle (Direction b)**: Lateral stretching occurs along the non-principal axis of the LM path. Within the 50% strain region, the sensor's unique architecture activates a strain decoupling mechanism, ultimately resulting in a negative GF response.

Length Change: The LM sensor architecture design converts external tensile stress into channel flattening rather than elongation along the LM path. Experimental observations indicate minimal effective length change along the LM path: $\frac{\Delta L}{L_{0}}\approx0$.

Cross-sectional Area Change: Macro-scale lateral stretching causes the LM channel to widen laterally (W2> W), significantly increasing the cross-sectional area A of the LM path: $\frac{\Delta A}{A_{0}}\gg0$. In summary,

$$\frac{\Delta R}{R_{0}}<0$$

Additionally, regarding Figure 1d, it illustrates the zero-point drift of the FOCUS and LM sensors, specifically: the effect of temperature on the sensors when no strain is applied. FOCUS: Remains near the zero line, indicating the FOCUS sensor exhibits excellent zero-point temperature stability. LM sensor: Shows an upward slope as temperature increases, indicating the LM sensor exhibits significant zero-point drift.

**Supplementary Note 2: The signal amplification principle of the Wheatstone bridge.**

This is essentially its unique differential measurement mechanism, which converts resistance changes in all bridge arms into voltage changes in the same direction that can be added together, thereby maximizing the output signal. Let's use a formula to clearly illustrate this. Assume that the bridge consists of four sensors S1, S2, S3, and S4, with resistors R1, R2, R3, and R4, respectively. The input voltage is $\text{V}_{\mathrm{in}}$, and the output voltage is $\text{V}_{\text{out}}$.

1. Bridge Output Voltage Formula

A Wheatstone bridge can be viewed as two voltage dividers connected in parallel. Its output voltage, $\text{V}_{\text{out}}$, is the difference between the two voltage divider output voltages:

$$\text{V}_{\text{out}}\text{=}\text{V}_{\text{in}}\text{⋅}\left( \frac{\text{R}_{\text{2}}}{\text{R}_{\text{1}}\text{+}\text{R}_{\text{2}}}\text{ -}\frac{\text{R}_{\text{4}}}{\text{R}_{\text{3}}\text{+}\text{R}_{\text{4}}} \right)$$

2. Sensor Resistance Change

About FOCUS design, when we apply tensile strain, the sensor's resistance changes as follows: Stretching the bridge arm (R1, R3): The resistance increases. Compressing the bridge arm (R2, R4): The resistance decreases. $\text{R}_{\text{0}}$ is the initial resistance, $\text{Δ}\text{R}$ is the resistance change.

3.Formula Derivation: Unveiling the Secret of Forward Superposition Now, substitute the changed resistance values into the output voltage formula:

$$\text{V}_{\text{out}}\text{=}\text{V}_{\text{in}}\text{⋅}\left( \frac{\text{(}\text{R}_{\text{0}}\text{+Δ}\text{R}\text{)}}{\text{(}\text{R}_{\text{0}}\text{-Δ}\text{R}\text{)+(}\text{R}_{\text{0}}\text{+Δ}\text{R}\text{)}}\text{-}\frac{\text{(}\text{R}_{\text{0}}\text{-Δ}\text{R}\text{)}}{\text{(}\text{R}_{\text{0}}\text{+Δ}\text{R}\text{)+(}\text{R}_{\text{0}}\text{-Δ}\text{R}\text{)}} \right)$$

Simplifying the denominators, both equal 2:

$$\text{V}_{\text{out}}\text{=}\text{V}_{\text{in}}\text{⋅}\left( \frac{\text{R}_{\text{0}}\text{+Δ}\text{R}}{\text{2}\text{R}_{\text{0}}}\text{-}\frac{\text{R}_{\text{0}}\text{-Δ}\text{R}}{\text{2}\text{R}_{\text{0}}} \right)$$

Merge molecules:

$$\text{V}_{\text{out}}\text{=}\text{V}_{\text{in}}\text{⋅}\left( \frac{\text{(}\text{R}_{\text{0}}\text{+Δ}\text{R}\text{)-(}\text{R}_{\text{0}}\text{-Δ}\text{R}\text{)}}{\text{2}\text{R}_{\text{0}}} \right)$$

Simplify the numerator further:

$$\text{V}_{\text{out}}\text{=}\text{V}_{\text{in}}\text{⋅}\left( \frac{\text{2Δ}\text{R}}{\text{2}\text{R}_{\text{0}}} \right)$$

Finally, we get:

$$\text{V}_{\text{out}}\text{=}\text{V}_{\text{in}}\text{⋅}\frac{\text{Δ}\text{R}}{\text{R}_{\text{0}}}$$

The final formula clearly demonstrates that despite the physical changes in the bridge arms occurring in opposite directions (one resistance increases, the other decreases), in this specific configuration of the Wheatstone bridge, they both contribute positively to the final output voltage. The change in output voltage is proportional to $\text{Δ}\text{R}$ and is four times greater even more than the change in a single bridge arm. Due to the differing sensitivity of the LM sensor under dual-axis tensile strain (with direction b decreasing more rapidly, Figure 3b), the FOCUS sensor exhibits over five times the sensitivity compared to the LM sensor. This is the core innovation of the FOCUS design. By transforming two opposing physical changes into a synergistic electrical enhancement, it achieves ultra-high sensitivity unattainable by traditional flexible sensors.
